# Covalent Organic Polymers and Frameworks for Fluorescence-Based Sensors

**DOI:** 10.1021/acssensors.1c00183

**Published:** 2021-04-07

**Authors:** Tina Skorjanc, Dinesh Shetty, Matjaz Valant

**Affiliations:** † Materials Research Laboratory, University of Nova Gorica, Vipavska 11c, 5270 Ajdovscina, Slovenia; ‡ Department of Chemistry & Center for Catalysis and Separations (CeCaS), Khalifa University of Science and Technology, P.O. Box 127788, Abu Dhabi, United Arab Emirates; § Institute of Fundamental and Frontier Sciences, University of Electronic Science and Technology of China, Chengdu 610054, China

**Keywords:** fluorescence, sensors, covalent organic polymers, covalent organic frameworks, quenching, ions, explosives, amines, biological molecules, enantiomers

## Abstract

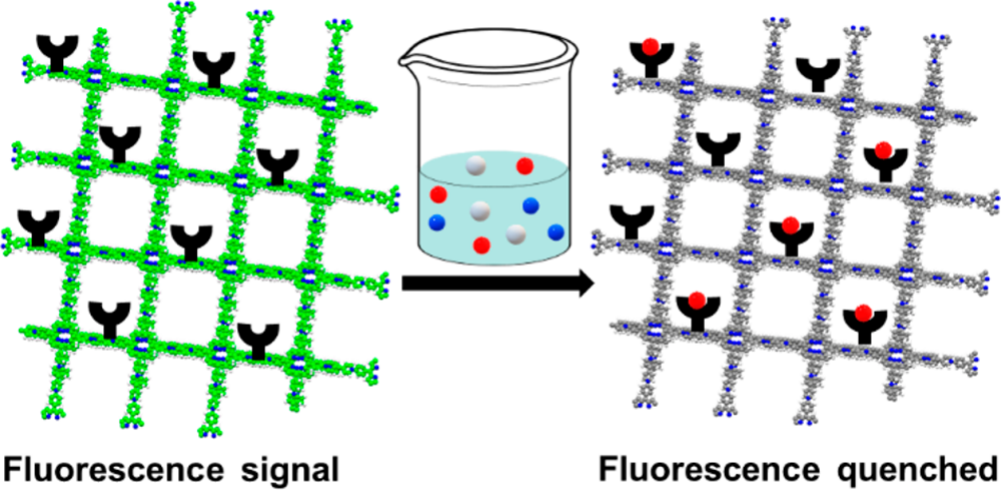

Following the advancements and diversification in synthetic strategies for porous covalent materials in the literature, the materials science community started to investigate the performance of covalent organic polymers (COPs) and covalent organic frameworks (COFs) in applications that require large surface areas for interaction with other molecules, chemical stability, and insolubility. Sensorics is an area where COPs and COFs have demonstrated immense potential and achieved high levels of sensitivity and selectivity on account of their tunable structures. In this review, we focus on those covalent polymeric systems that use fluorescence spectroscopy as a method of detection. After briefly reviewing the physical basis of fluorescence-based sensors, we delve into various kinds of analytes that have been explored with COPs and COFs, namely, heavy metal ions, explosives, biological molecules, amines, pH, volatile organic compounds and solvents, iodine, enantiomers, gases, and anions. Throughout this work, we discuss the mechanisms involved in each sensing application and aim to quantify the potency of the discussed sensors by providing limits of detection and quenching constants when available. This review concludes with a summary of the surveyed literature and raises a few concerns that should be addressed in the future development of COP and COF fluorescence-based sensors.

The idea of electron pair sharing between neighboring atoms, which is central to the definition of a covalent bond, was first introduced by Gilbert N. Lewis in 1916 when he presented the Lewis dot structures of covalent compounds.^1,2^ Eleven years later, the covalent bond was also defined quantum mechanically in terms of atomic orbital overlap by Walter Heitler and Fritz London.^3^ In the same decade, with the development of organic synthesis, the first covalently linked polymers were produced^4,5^ and the field of polymer science started to flourish. Unlike most electrostatic bonds, including coordination, hydrogen, and hydrophobic bonds, covalent bonds exhibit high bond strengths and can thus be applied in the synthesis of chemically more stable and robust materials. It is therefore not surprising that nylon, polyethylene, polystyrene, polyamide, and other products of covalent polymerizations found countless uses in everyday life.^6^

More recently, insoluble and porous covalent polymers have been praised for their extensive surface areas, which can interact favorably with other molecules through noncovalent interactions, a feature particularly useful in the fields of gas separation and storage, pollutant removal, drug delivery, catalysis, energy conversion, and sensing. These materials have been known under various names, but in the current review we primarily resort to the term covalent organic polymers (COPs).^7^ Typically, their backbones consist of lightweight elements such as C, H, N, O, F, and S. For specific applications that require metal ions, these can be introduced through coordination bonding. In 2005, these covalent polymeric materials were supplemented by covalent organic frameworks (COFs).^8^ The latter are characterized by the presence of dynamic covalent bonds, structural long-range order, and crystallinity.^9^ COFs diffract X-rays, which allows for the precise structure determination by the modeling of the experimental diffraction patterns. Such periodicity enables experimental and in silico molecular-level investigation of various phenomena, eliminates batch-to-batch variations in cross-linking density, and facilitates precise fine-tuning of the structure by postsynthetic modification.

Crystalline COFs and COPs can be designed in such a way that the extended π-conjugation in their backbone results in useful light-emitting properties. In comparison with traditional small-molecule chemosensors, these materials are insoluble in water and common organic solvents, which facilitate their separation, regeneration, repeated use, and incorporation into devices. Periodic structures allow introduction of specific target sites to increase the specificity. Furthermore, they possess large surface areas that can interact with targets, and their electronic and photophysical properties are tunable. While small-molecule chemosensors may also be noncovalently embedded into various matrices to enhance their stability, these systems may face the issue of leaching that COPs and COFs avoid on account of their covalent bonding. All of these properties make COPs and COFs promising sensors for a range of analytes, particularly those that interact with highly specialized environments such as enantiomers.^10^ These optical sensors can be based on different transduction mechanisms, including absorption, resonance, and fluorescence.^11^ Because fluorescence detection is significantly more sensitive than absorbance and can thus detect lower analyte concentrations,^12^ there is a push in the literature to design these kinds of materials for the detection of various analytes.

In this review, we first discuss the physical basis of fluorescence using the Jablonski diagram to illustrate the processes of excitation and emission. We also detail the various fluorescence mechanisms that are found in the existing fluorescence-based COP and COF sensors, including internal charge transfer (ICT), resonance energy transfer (RET), photoinduced electron transfer (PET), and aggregation-induced emission (AIE). In the next major section of the review, we describe the progress in the synthesis and performance of the relevant fluorescent sensors, primarily discussing the reports covering the past ten years. The synthetic approaches in these reports range from several kinds of C–C and C–N coupling reactions, boronic acid dehydration, azine, hydrazone, imine, imide and amide chemistries to triazine formation. We focus on various analytes, for instance explosives, metal cations, biological molecules, gases, amines, iodine, solvents, volatile organic compounds (VOCs), enantiomeric compounds, and anions. The current review concludes by summarizing the findings obtained herein and providing some future perspectives on the development of fluorescent COPs and COFs for sensing applications.

## Physical Basis of Fluorescence and Its Detection

**Figure 1:**
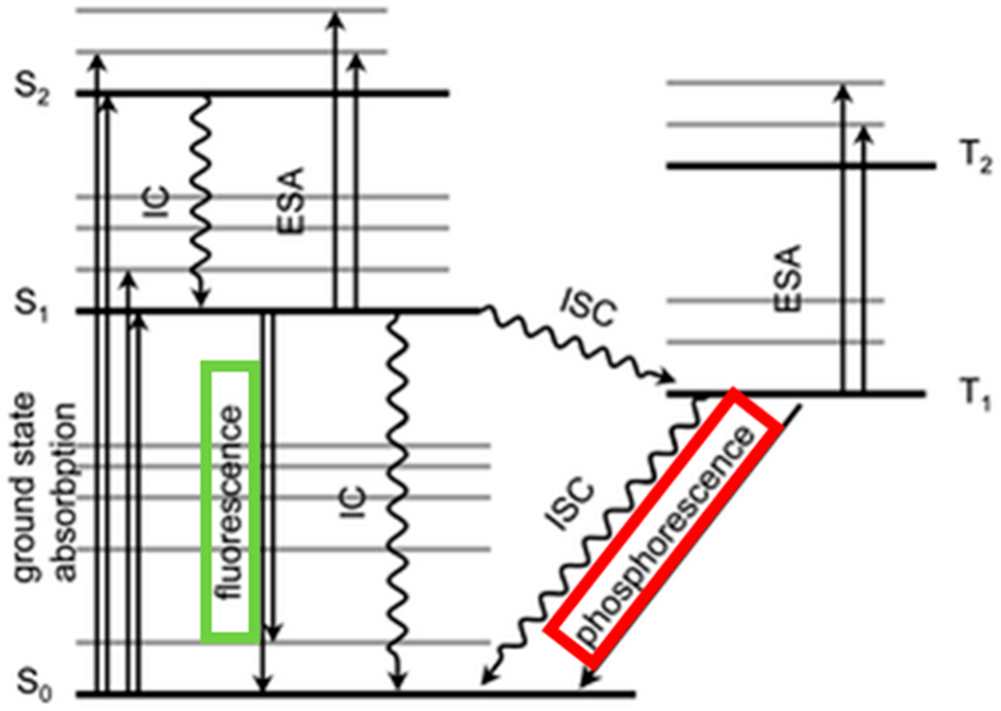
Jablonski diagram illustrating the phenomena of fluorescence and phosphorescence: IC, internal conversion; ESA, excited state absorption; ISC, intersystem crossing. Reproduced from ref (13) with permission from the PCCP Owner Societies. Copyright 2003.

The phenomena of fluorescence and other types of radiative decay are commonly explained with the aid of the Jablonski diagram (Figure 1).^13^ As a molecule absorbs energy, it is promoted from the ground state (S_0_) to the excited state (S_1_). It is then subjected to various collisions with surrounding molecules, which force it to lose some energy and step down the ladder to the lowest vibrational state of the electronically excited state (S_1_).^14^ From there, the molecule returns to the electronic ground state S_0_ while emitting energy in the form of light as a fluorescent signal. Fluorescence usually occurs at longer wavelengths (higher frequencies) than the incident radiation because some energy is lost to the surroundings in the form of nonradiative decay. This observation is known as the Stokes shift. If a molecule contains a moderately heavy atom (which is sometimes the case in COPs and COFs), such an atom may induce strong spin–orbit coupling and force the molecule from the excited S_1_ state into the triplet T_1_ state in a process known as intersystem crossing (ISC). This state is significantly more stable and the molecule may remain trapped in it for several seconds or even minutes. However, it is still able to emit light weakly and gradually return to the ground state. This phenomenon of light emission is known as phosphorescence,^14^ but due to its long lifetime it is not generally used in sensors.

## Fluorescence Mechanisms

**Figure 2:**
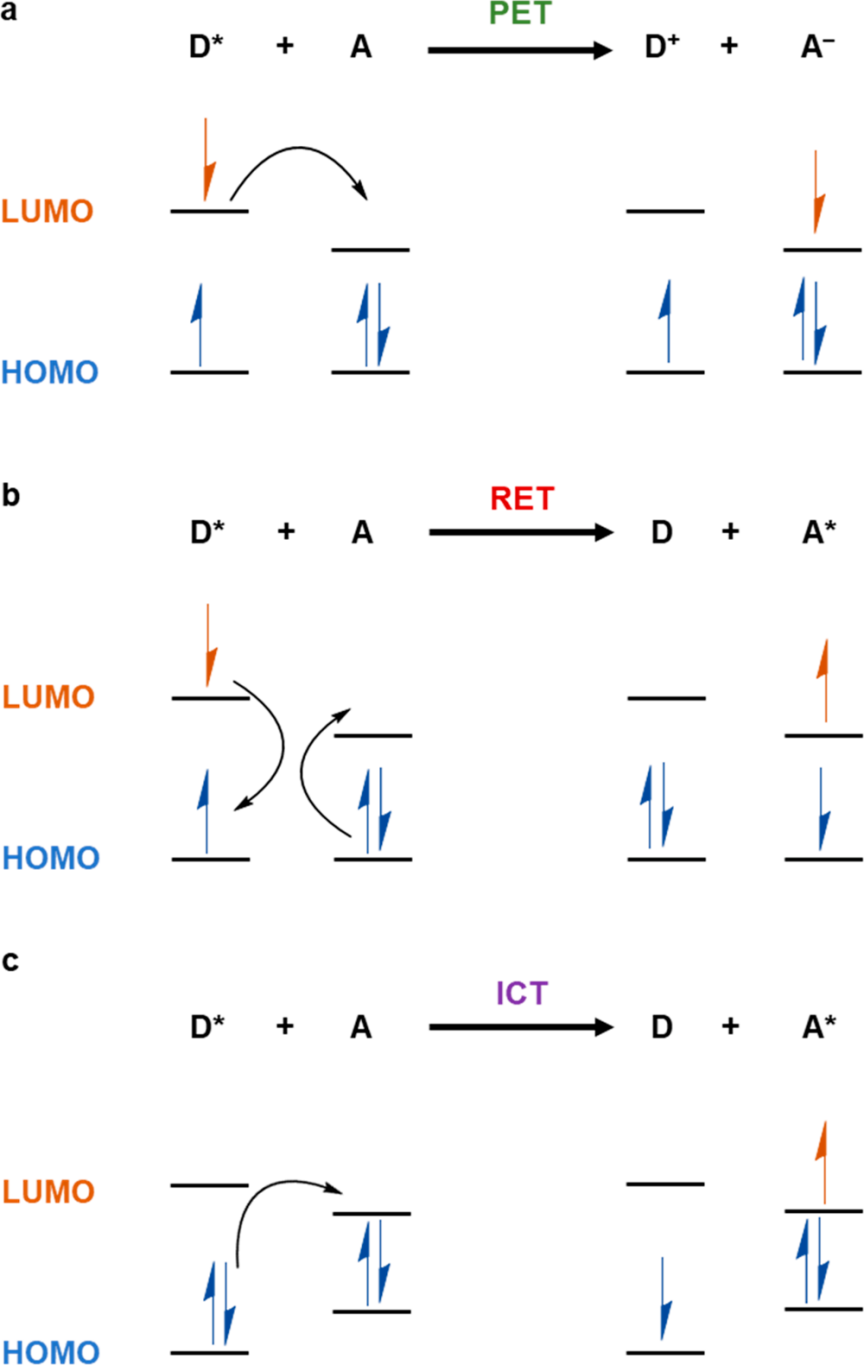
Schematic representation of the three main fluorescence mechanisms: photoinduced electron transfer (PET; a), resonance energy transfer (RET; b), and intramolecular charge transfer (ICT; c).^21^ D = donor; A = acceptor.

Multiple types of fluorescence mechanisms have been identified in donor and acceptor fluorophores in COPs and COFs sensors (Figure 2).^15^ In PET, the electron donor and acceptor form a charge transfer complex, which relaxes from the excited to the ground state with or without emission of a photon. In the former case, a fluorescence signal is observed and the extra electron on the acceptor is returned to the donor in the last step of the process (Figure 2a). This type of mechanism is commonly encountered in COP and COF sensors for heavy metal ions, where the excited electrons of the materials are transferred to the partly filled d-orbitals of the metal ions.^16^ In RET an initially excited donor molecule relaxes to the ground state while simultaneously transferring energy to the acceptor molecule, which is in turn promoted to the excited state (Figure 2b). This process requires that there be overlap between the emission spectrum of the donor fluorophore and the absorption spectrum of the acceptor and is commonly seen in DNA sensors.^17^ The term Förster resonance energy transfer (FRET) is often encountered in the literature,^18^ and it refers to the same kind of a physical process which occurs specifically at the Förster radius, a distance between the fluorophores where the energy transfer efficiency is 50%. In ICT, a single fluorophore must contain both an electron donating and an electron accepting group, such as a phenyl and a triazine ring, respectively. Photoinduced excitation can increase charge separation in the fluorophore, and subsequent events depend on the polarity of the solvent. A polar solvent stabilizes the species with the charges separated. In contrast, in a nonpolar solvent, the species without charge separation may have the lowest energy (Figure 2c).^19^ This type of the mechanism could be used in a fluorescent sensor, which contains electron donating and accepting groups, but its ICT is restricted by the presence of the analyte, which interacts with one of the two groups.^20^

## Fluorescence Spectroscopy of Covalent Polymeric Sensors: Quenching, Enhancement and Shifts

When an analyte is added to a fluorescence-based sensor, any of the above mechanisms may be triggered, which typically manifests in one of the three changes in the fluorescence spectrum of the fluorophore: (i) the intensity decreases which is referred to as turn-off fluorescence or quenching, (ii) the intensity increases, which is known as turn-on fluorescence or enhancement, or (iii) the maximum intensity shifts to a different wavelength.

The fluorescence quenching is a widely studied theory in the literature,^22^ which can occur as a result of diffusive encounter (i.e., dynamic quenching) or due to the formation of a complex (i.e., static quenching). These two processes can be distinguished on the basis of their lifetimes (τ). In static quenching, the fluorescence lifetime remains unchanged if the concentration of the quencher (e.g., an analyte) is increased because any remaining fluorophore moieties, which do not complex with the quencher, retain their original fluorescence lifetime. Thus, τ_0_/τ = 1. In dynamic quenching, by contrast, increasing concentration of the quencher reduces the fluorescence lifetime. This can be explained by the fact that the greater presence of the analyte increases the chance of a collisional encounter with the fluorophore. Therefore, τ_0_/τ = *F*_0_/*F*. Both types of quenching can be described by Stern–Volmer equations, which allow for the determination of the quenching constants. For further details on these constants, the reader is advised to refer to related reviews.^23−25^ These types of quenching as well as other mechanisms of fluorescence are further illustrated by specific examples in the next major section of the review.

The materials discussed herein have been divided into groups by the type of the analyte they can detect. Within each group, materials are compared to each other by several metrics, including the limits of detection (LODs; the lowest quantity of an analyte that can be distinguished from the absence of that analyte, or a blank) and Stern–Volmer quenching constants, where applicable. The materials are also evaluated and compared among each other in terms of their selectivity and recyclability.

## Sensors for Heavy Metals

Numerous heavy metals are naturally present in the Earth’s crust, and many of them (e.g., Fe, Cu, Co, Mg, Mo, Cr, Se, Mn, Ni, and Zn) are required for the proper functioning of the human body. However, excess ingestion of these metals can have negative effects on human health. In addition, certain other metals, such as Hg, Cd, Pb, As, and Ag, are toxic to humans in even minute concentrations.^26^ Therefore, it is instrumental to design materials, which can detect the presence of these potentially dangerous metals in drinking water, food, and the environment. In this subsection, we comprehensively review fluorescence COPs and COFs for the detection of Hg^2+^, Fe^3+^, Cu^2+^, Cr^3+^, and a few mixed metal cation sensors. A typical mechanism involved in these types of sensors is an electron transfer from the excited material to the partially filled d-orbitals of the metal cations through the PET mechanism. Regardless of the metal, the strategy of having predesigned metal chelation sites in the structure almost always results in higher specificity and lower LODs.

### Mercury

Mercury is a highly toxic metal, which is known to cause mercury poisoning and can even lead to the deadly Minamata disease. Thus, the maximum allowed concentration in drinking water is 2 ppb, 2 μg L^–1^, or ≈10 nM. Hg^2+^ ions are known to have an affinity for S-containing groups.^27^ While Hg^2+^ sensors without these moieties have also been reported, it is primarily those with free thiol, thioether, and carboxyhydrazide groups that reach lower detection limits and greater selectivity (Table 1). For example, the P1 COP sensor for Hg^2+^ based on the turn-on fluorescence was prepared by incorporating a small-molecule fluorophore 5,5-difluoro-1,3,7,9-tetramethyl-10-phenyl-5*H*-dipyrrolodiazaborinine (BODIPY) into a covalent polymer.^28^ The BODIPY dye is nonfluorescent in the original state on account of a rapid C=N isomerization that is perturbed when Hg^2+^ forms a coordination bond with two neighboring dye moieties. The initially non-emissive material starts to emit light in the presence of the metal cations; however, the detection limit for Hg^2+^ is rather high at 0.37 μM.

**Table 1:** COPs and COFs Used As Heavy Metal Ion Sensors

material	metal ion	sensing mechanism	*K*_SV_ (M^–1^)	LOD	ref
COF-LZU8	Hg^2+^	quenching	n/a	25 ppb	(29)
TNPP	Hg^2+^	quenching	3.78 × 10^5^	22.8 ppb	(30)
NOP-28	Hg^2+^	PET (quenching)	3.7 × 10^4^	12 ppb	(16)
TFPPy-CHYD	Hg^2+^	PET (static quenching)	n/a	17 nM	(31)
P1	Hg^2+^	turn-on fluorescence	n/a	0.37 μM	(28)
COP-401-COOH	Fe^3+^	PET (quenching)	8.4 × 10^4^	n/a	(26)
COP-64	Fe^3+^	PET (quenching)	1.10 × 10^5^	n/a	(27)
COP-9	Fe^3+^	ACQ	1.7 × 10^4^	mM range	(34)
COP-1	Fe^3+^	PET (static quenching)	n/a	0.42 μM	(33)
TPA-COP	Fe^3+^	chelation-induced turn-on fluorescence	n/a	0.43 μM	(37)
COP-100	Fe^3+^	collisional quenching	2.97 × 10^4^	0.245 μM	(42)
Bth-Dma COF	Fe^3+^	quenching	2.3 × 10^4^	0.17 μM	(38)
COF-JLU3	Cu^2+^	PET (quenching)	3.8 × 10^4^	0.31 μM	(40)
COPs-DT	Cu^2+^	PET (quenching)	n/a	0.076 μM	(41)
Salen-COP	Cu^2+^	CHEQ	n/a	0.545 μM	(43)
PI nanonsheets	Cr^3+^	Quenching	n/a	n/a	(44)
TFPT-BTAN-AO	UO_2_^2+^	PET (quenching)	n/a	6.7 nM	(32)
CorMeO–COF	Cr^3+^	turn-on fluorescence	n/a	0.85 μM	(45)
Salen-COP	Al^3+^	turn-on fluorescence, PET	n/a	0.248 μM	(43)
Salen-COP	Fe^3+^	CHEQ	n/a	0.140 μM	(43)

**Figure 3:**
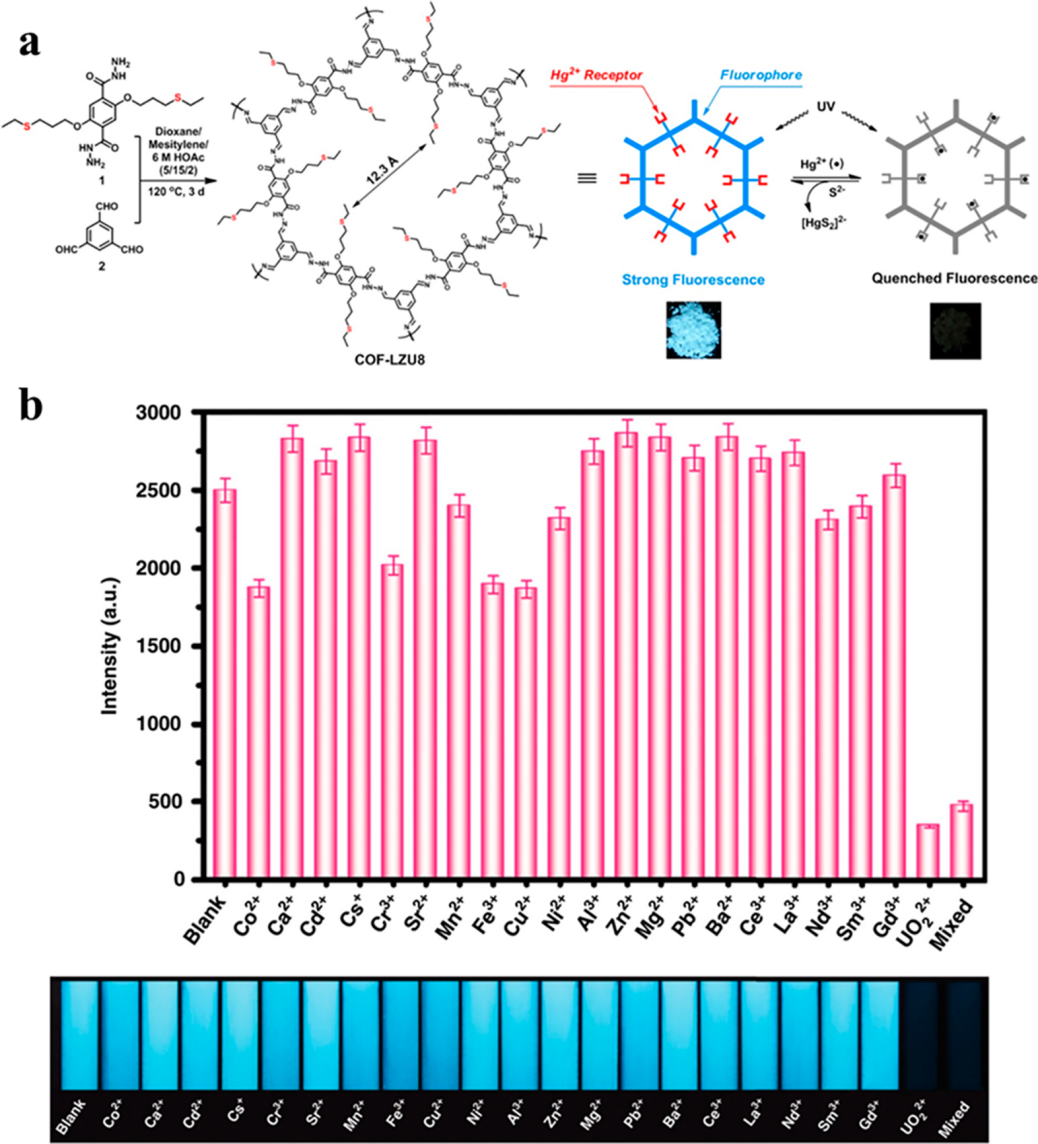
Sensing of heavy metal ions. (a) Design strategy for COF-LZU8 and the fluorescence quenching mechanism triggered by the addition of Hg^2+^ ions. Reproduced with permission from ref (29). Copyright 2016 American Chemical Society. (b) Selectivity of TFPT-BTAN-AO for UO_2_^2+^. Reproduced with permission from ref (32). Copyright 2020 The Authors under Creative Commons Attribution 4.0 International license (https://creativecommons.org/licenses/by/4.0/).

The very first manuscript on fluorescent COFs for metal ion detection was published by Wang et al. in 2016, and it triggered the development of diverse COF-based detection systems. In this work, the authors constructed COF-LZU8 with thioether functionalities on the starting hydrazide, which produced a polymeric structure with thioethers pointing both toward and away from the pore interior (Figure 3a).^29^ The COF exhibited a strong fluorescence signal upon excitation at 390 nm, which was quenched by the addition of Hg^2+^ ions in acetonitrile. The COF possessed good selectivity as it preferentially removed Hg^2+^ even in the presence of 2 equiv of various competitive metal ions. The LOD was 25 ppb. A similar LOD (22.8 ppb) was also observed for a triarylamine-based COP synthesized through Suzuki coupling and postsynthetically modified with thiosemicarbazone to introduce Hg^2+^ chelation sites.^30^

Reversibility of Hg^2+^ binding, a feature integral to reuse of the sensor, was not considered in COF-LZU8 (Figure 3a),^29^ but a later manuscript found it to be nearly irreversible.^31^ It was therefore urgent to design materials that will serve not only as chemodosimeters but rather as reversible Hg^2+^ sensors. Sulfide-bridged polytriazine nanospheres NOP-28 were fully reversible and retained specificity among 11 different metal cations.^16^ The metal ion detection was based on fluorescence quenching at 415 nm ascribed to the electron transfer from the COP to the formed guest metal complexes, or metal ion-induced polymer chain aggregation. Their LOD in ethanol was still rather high at 12 ppb.

The issue of reversibility was also addressed in the TFPPy-CHYD COF where the thiol chelation sites were replaced by carbohydrazide linkages.^31^ This material reached a low LOD of 17 nM or 3.4 ppb, which is almost sufficient for sensing Hg^2+^ in drinking water. The pyrene–carboxyhydrazine conjugate was also praised for its fast response time (2 s), selectivity for Hg^2+^ in the presence of other cations and anions at five times higher concentrations in DMF, and facile regeneration ability with Na_2_S. This work thus represents a monumental step in the direction of sensitive, reversible Hg^2+^ COF sensors.

A few aspects of COPs and COFs mercury sensors remain unaddressed. First, organic solvents are used to dissolve Hg^2+^ while sensing in water would be the most relevant in environmental and health applications. This is likely due to the interference of highly polar water molecules in the fluorescence properties of the synthesized materials. Second, all of the discussed examples focus on inorganic Hg^2+^ detection. Although this species is toxic, it is the organic mercury such as methyl mercury, or [CH_3_Hg]^+^, that is even more hazardous. These species are formed when Hg^2+^ comes into contact with bacteria in water or soil, and its toxicity is particularly problematic due to its bioaccumulation. Therefore, future sensor design should not neglect [CH_3_Hg]^+^, although its handling and storage in a laboratory setting may be challenging.

### Iron

Iron is an essential element in various metabolic processes, including oxygen uptake into red blood cells and its transfer, and DNA synthesis. However, if present in higher amounts in biological organisms, it can lead to disease, so iron detection is of importance. The maximum allowed concentration of Fe^3+^ in drinking water is 5.4 μM, or ≈302 ppb. Similar to Hg^2+^ sensors, Fe^3+^ sensors also commonly incorporate metal cation chelation sites that lower the LODs.^33^ For example, Yamamoto cross-coupling produced a series of COPs (COP6–9) with LODs in the mM range. These materials were interesting for the absorption competition quenching (ACQ) mechanism for Fe^3+^ sensing.^34^ In DMF, both the COP and the Fe^3+^ ions absorb light in the 250–400 nm window, so upon excitation the two species compete for the light energy. For that reason, the presence of Fe^3+^ reduces the excitation of the COP and quenches the fluorescence signal. Interestingly, other divalent metal cations exhibit smaller absorption spectral overlap with the COP, so they are less able to quench the fluorescent signal, ultimately leading to selectivity for Fe^3+^.

A range of chelation sites can be incorporated into polymeric materials to make more efficient Fe^3+^ sensors. For instance, -COOH and -SO_2_Cl functionalities introduced into conjugated COPs synthesized through Yamamoto cross-coupling (e.g., COP-401-COOH and COP-64) have been reported for Fe^3+^ sensing.^35,36^ These modifications resulted in dynamic Stern–Volmer constants of 8.4 × 10^4^ and 4.3 × 10^4^ M^–1^, respectively, which is an order of magnitude higher than the parent, nonfunctionalized COP. In another study, Sonogashira–Hagihara^37^ cross-coupled TPA-COP contained the tridentate Schiff base sites able to selectively coordinate Fe^3+^ ions. Similar to the Hg^2+^ turn-on sensor discussed above,^28^ Fe^3+^ induces rigidity and suppresses PET from the imine N to the excited moiety, turning on the fluorescence. The authors refer to this phenomenon as chelation-induced turn-on fluorescence.

A particularly innovative report constitutes the Bth-Dma COF, the first example of a luminescent COF with predesigned O–N–O′ chelation site for the detection of metal ions in water. The O–N–O′ chelation sites in this hydrazone-linked COF participate in fluorescence quenching upon addition of Fe^3+^ ions.^38^ XPS studies on the Fe 2p and N 1s orbitals further confirmed the chelation process. The LOD in this COF is low at 0.17 μM, and the Stern–Volmer quenching constant is *K*_SV_ = 2.3 × 10^4^ M^–1^, which is comparable to other COPs, COFs, and MOFs. Importantly, these values were obtained in water rather than in an organic solvent. Therefore, the results of this report may be more applicable to real-life sensing applications in environmental, food, and health sciences.

### Copper

Copper is an essential element for the human body. Enzymes utilize it in energy generation, neurotransmitter and pigment synthesis, and epigenetic modification. Its hyper-accumulation in biological fluids and tissues can, however, lead to diseases such as Wilson and Meknes.^39^ An azine-linked COF-JLU3 with a fluorescence half-life of 1.5 ns was utilized for selective detection of Cu^2+^ in THF.^40^ The mechanism involved PET from the COF to the d-orbitals of the Cu^2+^ ions that coordinated to the framework through Lewis acid–base interactions with the OH side functional groups and azine linkages. The LOD of this system was 0.31 μM, and the Stern–Volmer quenching constant was calculated to be 3.8 × 10^4^ M^–1^. In another study, four times lower LOD (0.076 μM) was achieved with imine-linked COPs-DT sensors with the same kind of sensing mechanism.^41^ Given that the surface areas of the two materials are comparable (456 and 540 m^2^ g^–1^), better performance of COPs-DT might be attributed to the solvent (isopropanol), or the strength of chelation. Notably, this material exhibited a good level of selectivity among 16 metal cations tested and could be regenerated by adding a strong competitive metal chelating agent (EDTA) without compromising the sensitivity. COPs and COFs for sensing Cu^2+^ in water are yet to be explored in greater detail and so are those for sensing copper species with oxidation states other than 2^+^.

### Uranium

With increasing dependence on nuclear power plants for the generation of electricity, the issue of radioactive waste is more significant than ever before. Materials are being developed to detect uranium and its oxides, such as the TFPT-BTAN-AO COF generated through the Knoevenagel condensation and postsynthetically modified by amidoximation (Figure 3b).^32^ This work is truly remarkable for several reasons: (1) it utilizes Knoevenagel condensation, a lesser-known chemistry in the COF synthesis, (2) postsynthetic amidoximation introduces a large number of -OH and -NH_2_ functional groups into the network, which serve as exclusive binding sites for UO_2_^2+^ among 21 metal cations tested, and (3) upon excitation at 270 nm in water, the LOD is 6.7 nM, which is well below the WHO contamination limit for UO_2_^2+^ in drinking water (63 nM). XPS results show that UO_2_^2+^ binds to the N atoms of the NH_2_ groups and the O atoms (O 1s and N 1s peaks shift 0.20 and 0.22 eV to higher energies, respectively, and new U–N and U–O peaks appear at 401.1 and 531.3 eV, respectively). As such, this work presents a COF sensor of the highest merit: it is operational in water, exhibits high selectivity and LOD below the allowed concentration in water, and demonstrates an innovative synthetic approach. The challenge now lies in scaling up the synthesis of the material and optimizing its production cost.

### Groups of Metal Cations with Common Physical Properties

Some sensors for heavy metal cations were not specific to a particular metal such as the ones presented above but rather exhibited selectivity based on common properties of metal ions, such as valency and/or oxidation number,^43,45^ atomic mass,^46^ and/or metals with filled d-orbitals.^47^ The utility of these systems depends on their purpose; they might be very helpful when the presence of any kind of heavy metal is of interest but less so when our interest is in a particular metal ion. In most cases, the materials contain main-chain or side functional groups that form different interactions with metal cations depending on the type of metal. For instance, an azo-linked conjugated COP exhibited fluorescence quenching when exposed to heavy metals but showed no response to light metals such as Na^+^ and Ca^2+^. This difference was rationalized by the hard acid nature of Na^+^, K^+^, and Ca^2+^, which leads to a low affinity for aromatic systems and azo groups.^46^

One of the most interesting examples of COFs showing distinct fluorescence responses to different heavy metal ions is the corrole-based CorMeO–COF.^45^ This material can be used as a rather general heavy metal sensor, as it exhibits turn-on fluorescence in the presence of trivalent metal ions (Al^3+^, Cr^3+^, Ga^3+^, and Fe^3+^) in THF. The 2D COF had strong interlayer π–π stacking interactions, which were loosened in the presence of these trivalent metal ions, enhancing the fluorescence signal. By observing that an analogous porphyrin-based COF with a similar skeleton does not exhibit enhanced fluorescence in the presence of these metals, the authors conclude that the fluorescence phenomenon is a result of aggregation-induced quenching, which is common in bulk 2D-COF materials. The LODs were found to be in the low-μM concentration, which is within the range of maximum allowed limits. It is important to note that some trivalent heavy metals (Tb^3+^, La^3+^, Eu^3+^, and Pr^3+^) among those tested induce only a little fluorescence enhancement.

In general, studies aiming at detecting groups of metal cations should be supplemented by an investigation on the response to mixtures of heavy metals. It would be expected that the sensitivity for each individual metal would decrease in the presence of other metal ions, but the extent of the reduction would need to be carefully evaluated.

## Sensors for Explosives

For safety reasons, effective sensors for explosives are currently high in demand. It is therefore not surprising that a plethora of COPs and COFs sensors were designed for compounds such as picric acid or 2,4,6-trinitrophenol (TNP), 2,4,6-trinitrotoluene (TNT), 2,6-dinitrophenol (DNP), 2,6-dinitrotoluene (DNT), 2-nitrophenol (NP), and 2-nitrotoluene (NT; Table 2).^48^ Diverse chemical reactions have been utilized for the synthesis of nitroaromatics sensors, including nitrile trimerization into triazines,^49^ Sonogashira–Hagihara coupling,^50,51^ Yamamoto coupling,^52,53^ Suzuki coupling,^54^ Buchwald–Hartwig coupling,^55^ and imine^56^ and azo bond formations.^57^

**Table 2:** COPs Used as Nitro Explosive Sensors

material	explosive	sensing mechanism	*K*_SV_ (M^–1^)	LOD	ref
MAEC-PMA	TNP	static quenching	2.95 × 10^4^	93.3 nM	(59)
CMP-LS1	TNP	PET, RET	5.05 × 10^4^	n/a	(54)
CMP-LS2	TNP	PET, RET	3.70 × 10^4^	n/a	(54)
COP-612	TNP	static quenching	2.5 × 10^5^	n/a	(52)
COP-401	TNP	PET (dynamic quenching)	8.3 × 10^4^	<1 ppm	(53)
COP-301	TNP	PET (dynamic quenching)	2.6 × 10^5^	<1 ppm	(53)
SNW-1	TNP	quenching	9.4 × 10^4^	50 nM	(58)
A-NS	TNP	static quenching	8 × 10^5^	90 nM	(56)
PrTAPB-Azo-COP	TNP	static quenching	1.1 × 10^4^	n/a	(57)
COP-3	TNP	PET (static quenching)	1.45 × 10^4^	<1 ppm	(60)
DTF	TNP	quenching	2.08 × 10^3^	0.722 μM	(51)
LMOP-15	TNP	PET (quenching)	2.6 × 10^4^	0.33 μM	(61)
LPCMP2	TNT	PET	4.15 × 10^3^	3.64 μM	(55)
PCPDI	*o*-NP	ACQ, IFE	1.74 × 10^5^	17.2 pM	(49)
DP2A2	*o*-NP	PET (static quenching)	2.00 × 10^4^	1.5 nM	(50)

A range of COPs have been used for detecting nitro explosives, and this section only discusses the highlights of the literature that exists in the area. While some materials serve as sensors for nitro explosives in general,^58^ others are tailored to specific explosive compounds.^49,53^ Nitrophenols contain a polar hydroxyl group able to form hydrogen bonds that is lacking in nitrotoluenes, often leading to selectivity. For instance, the PCPDI triazine framework formed through trimerization of aromatic nitriles of *N*,*N*′-di(4-cyanophenyl)-3,4,9,10-tetracarboxylic diimide was selective for *o*-NP to a very low level of 17.2 pM.^49^ Other explosives, including DNT, *p*-NP, *m*-NP, and NT exhibited negligent quenching effect. This selectivity was explained by the formation of hydrogen bonds between *o*-NP, and O- and N-rich PCPDI and the absorption spectra overlap between the two. The hydrogen bonding is also responsible for the ACQ mechanism dominating the fluorescence response. Similarly, in a mixture of TNP, NT, DNT, *m*-DNB, *p*-DNB, and NP, Yamamoto-coupled conjugated COP-612 showed a strong preference for TNP.^52^

**Figure 4:**
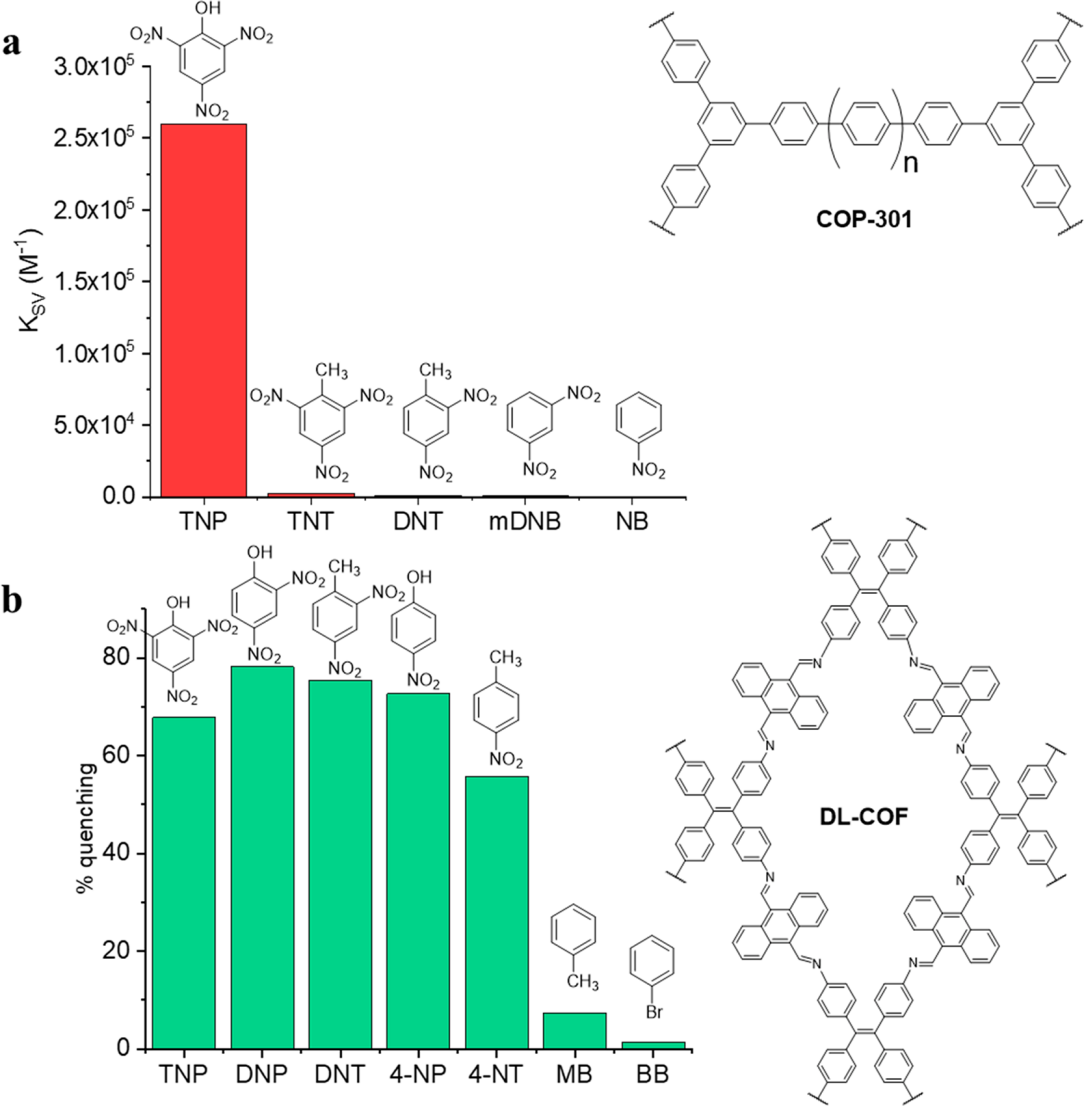
Selectivity of sensors for nitro explosives: (a) COP-301 sensor for a specific nitro explosive, TNP;^53^ (b) DL-COF, a general sensor for nitro explosives.^65^

A detailed study on the mechanism of explosives detection in COP-301, another Yamamoto-coupled COP, has found two major contributions to the selectivity for TNP: (i) the relative positions of HOMO and LUMO and (ii) the electron withdrawing effects of -NO_2_ functional groups (Figure 4a).^53^ The COP and the nitro explosives are aromatic, so they interact through π–π stacking interactions. Upon excitation, electrons in the conduction band of the COP are transferred to the LUMO of TNP, which results in quenching. Because of electron withdrawing effect of the nitro groups, TNP has the lowest LUMO, so its quenching ability is the highest.

In the realm of COFs, one should highlight the very first example of a sensor constructed from this class of materials. This was an azine-linked chemosensor for TNP reported in 2013, which was simultaneously the first COF linked by azine bonds (Table 3).^62^ The fluorescence signal of Py-azine COF at 522 nm (λ_ex_ = 470 nm) was quenched by the addition of up to 70 ppm TNP due to the static quenching phenomenon. For the same reasons of hydrogen bond formation by nitrophenols but not nitrotoluenes, Py-azine COF along with imide-linked TfpBDH COF,^63^ and PI-COF^64^ showed selectivity for TNP. One of the most potent COFs used for the detection of general nitro explosives is DL-COF (Figure 4b).^65^ This material was not selective for a particular explosive as it exhibited high Stern–Volmer constants for TNP, DNP, DNT, 4-NP, and 4-NT (Table 3). Selectivity experiments demonstrated that non-explosives (e.g., toluene or bromobenzene) induced very little fluorescence quenching.

**Table 3:** COFs Used as Nitro Explosive Sensors

material	explosive	sensing mechanism	*K*_SV_ (M^–1^)	LOD	ref
Py-azine COF	TNP	static quenching	7.8 × 10^4^	n/a	(62)
TfpBDH	TNP	PET (dynamic quenching)	2.6 × 10^4^	n/a	(63)
TFPC-NDA COF	TNP	static and dynamic quenching	2.48 × 10^5^	n/a	(66)
PI-COF	TNP	PET, IFE	1 × 10^7^	0.25 μM	(64)
3D-Py-COF	TNP	quenching	3.1 × 10^4^	n/a	(67)
TAPB-TFPB	TNP	static quenching	5.9 × 10^4^	n/a	(68)
DL-COF	TNP	static quenching	2.24 × 10^6^	57.31 nM	(65)
DL-COF	DNP	dynamic quenching	4.28 × 10^6^	46.50 nM	(65)
DL-COF	DNT	dynamic quenching	3.71 × 10^6^	57.32 nM	(65)
DL-COF	4-NP	dynamic quenching	3.18 × 10^6^	37.05 nM	(65)
DL-COF	4-NT	dynamic quenching	1.56 × 10^6^	50.52 nM	(65)

Given the plethora of COP and COF sensors for explosives, this class of analytes is probably the most suitable to compare the relative merits of the two classes of materials. Almost all of the reports present quenching of the fluorescence signal upon the addition of the analytes, so a comparison is even fitter. The data in Tables 2 and 3 suggest that both COPs and COFs can reach very low LODs, down to nM or pM levels. A major difference that we observe, however, is in the magnitude of the Stern–Volmer quenching constants. We note that the COFs can exhibit *K*_SV_ values on the order of 10^6^ to 10^7^, whereas most COPs reach a maximum of 10^5^. This suggests that long-range order in COFs contributes to greater degrees of quenching for a given analyte concentration and initial fluorescence signal. It is therefore expected that the concentration of the analyte would be more accurately calculated from an experimental fluorescence measurement in the case of COFs.

## Sensors for Biological Molecules

As has been noted with other analytes, the structures of COPs and COFs can be adapted to form favorable interactions with disease markers (methylglyoxal, a marker for diabetes mellitus,^69^ the anthrax biomarker,^70^ or sialic acid, an ovarian cancer biomarker^71^), reactive oxygen species (hydroxyl radicals, ^•^OH),^72^ and antibiotics.^73^ In TpPa-1@LE, the imine linkages were deprotonated, which generated anionic N^**–**^ centers that could interact with methylglyoxal.^69^ The latter induced a shift in emission from 476 to 525 nm. The mechanism of fluorescence involved exciplexes (heterodimeric short-lived species generated in the excited state) that formed when the excited state TpPa-1@LE collided with methylglyoxal molecules and charge transfer occurred to form a luminescent intermediate. ^•^OH radicals, in contrast, transfer an electron to the COF-TpMA framework, forming an aggregate through π–π stacking and inducing fluorescence quenching.^72^ The FRET mechanism is involved in sensing tetracycline with Suzuki-coupled CMP-LS7 and CMP-LS8.^73^ These materials and tetracycline form π–π interactions through aromatic rings and hydrogen bonds.

**Figure 5:**
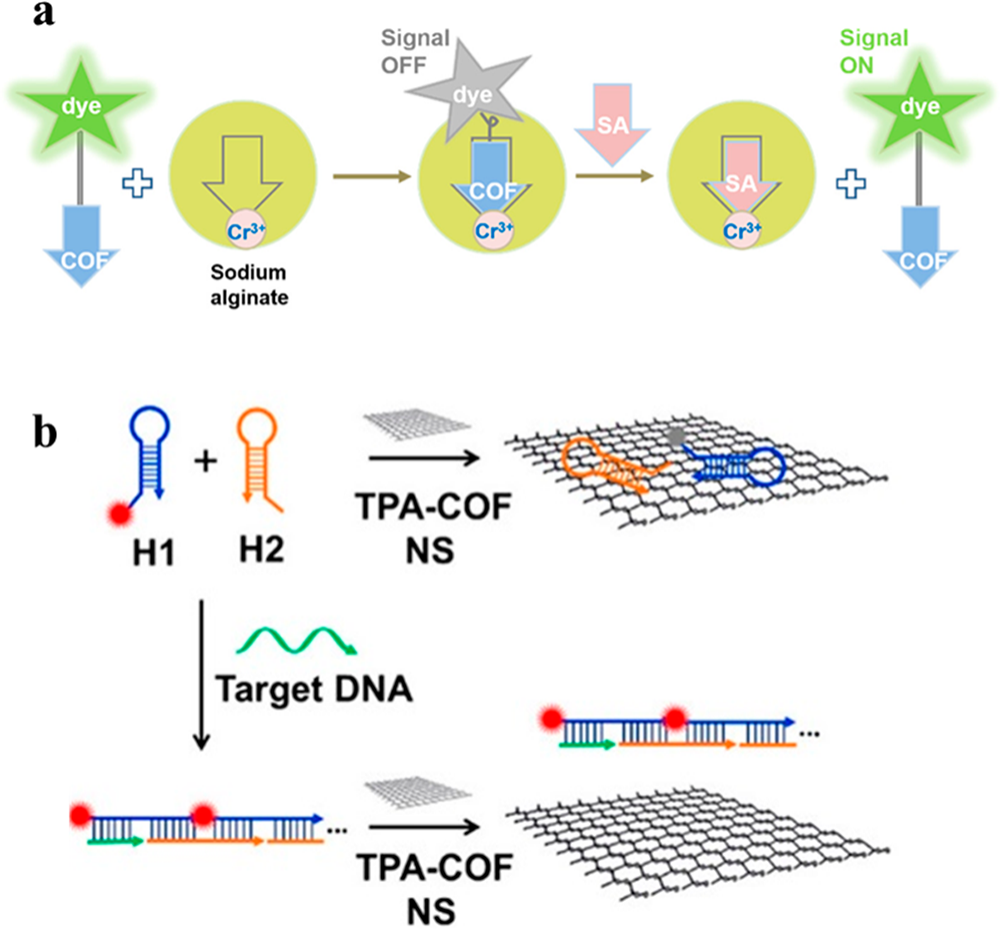
Operating principles of biomarker and DNA sensing platforms. (a) TpPa-1 turn-on sensor for sialic acid biomarker present in ovarian cancer. Reproduced with permission from ref (71). Copyright 2020 American Chemical Society. (b) TpTta COF sensor for DNA. Reproduced with permission from ref (75). Copyright 2016 American Chemical Society.

COF sensors for biological molecules often take advantage of incorporating metal ions that actively participate in the sensing process in various ways. First, the metal centers can act as electron acceptors. A sensor for levofloxacin used a COF as a solid support system for Eu^3+^ ions, which can bind strongly to β-diketone in levofloxacin through coordination bonds.^74^ Upon irradiation, the β-diketone substructure of the antibiotic enters the triplet state, from which an electron is transferred to the excited-state energy level of Eu^3+^. The light is emitted by the relaxation from that excited state. This example illustrates that the ordered nature makes COFs useful solid supports into which metal ions can be introduced in a controlled manner. The COFs thus prevent metal ion aggregation, facilitate separation from solution, and prevent leaching. The overall effect is a reproducible sensing behavior (Table 4). Second, the metal centers can give up their hydration to coordinate the analyte molecules (e.g., dipicolinic acid). This “antenna effect” results in turn-on fluorescence that originates from the metal center.^70^ Third, metal ions can participate in the indicator displacement assay (Figure 5a).^71^ Here, the metal center serves as a receptor of the analyte (sialic acid) and the dye-functionalized TpPa-1 COF as a turn-on fluorescence indicator.

**Table 4:** COP and COF Sensors for Biological Molecules

material	analyte	sensing mechanism	*K*_SV_ (M^–1^)	LOD	ref
CMP-LS7	tetracycline	IFE (quenching)	1.92 × 10^4^	0.94 μM	(73)
CMP-LS8	tetracycline	IFE (quenching)	8.86 × 10^4^	0.22 μM	(73)
Eu@TpPa-1	levofloxacin	PET, turn-on fluorescence	n/a	0.2 μM	(74)
Tb-COP	dipicolinic acid	turn-on fluorescence, antenna effect	n/a	13.5 nM	(70)
TpPa-1	sialic acid	turn-on fluorescence	n/a	7.08 nM	(71)
PF-DNA CPN	DNA	FRET	n/a	nM range	(17)
TpTta	DNA	FRET, turn-on fluorescence	n/a	3.7 nM	(75)
TPA-COF NSs	DNA	turn-on fluorescence	n/a	20 pM	(76)
COF-TpMA	^•^OH	static quenching	0.8281	n/a	(72)
TpPa-1@LE	methylglyoxal	exciplex formation	n/a	109.6 nM	(69)

DNA sensing has also been investigated with fluorescent COPs and COFs.^17,75,76^ In most cases, the sensors are attached covalently or noncovalently to the DNA strands complementary to the target DNA. Because of these complementary strands, the selectivity of these systems is very high, but it cannot be attributed to the COF. Similar to the Eu^3+^ ions in the antibiotic sensing,^74^ the role of the COF is primarily to serve as support or anchor point. For example, carboxylic acid-functionalized polyfluorenes were conjugated with a specific oligonucleotide and the thus-obtained system was used as a FRET sensor for specific complementary chains of DNA.^17^ In the presence of the target DNA and a FRET signal enhancer, the PicoGreen dye, quenching of the signal was observed at 426 nm and a new emission peak appeared at 530 nm. None of these changes were observed in the presence of noncomplementary DNA chains. In addition to being a solid support, COFs in DNA sensing can also be the quenchers of the DNA probe’s fluorescence (Figure 5b). In this case the probe is composed of a DNA strand complementary to the target and a fluorescent dye such as carboxyfluorescein^72^ or Texas red.^73^ Upon the addition of the target DNA, the probe detaches from the COF, interacts with the target DNA, and re-emits fluorescence through the FRET mechanism.^75,76^

In summary, some sensors for small biological molecules resemble the operating principles of explosives and heavy metals sensors by taking advantage of their own fluorescent nature and formation of noncovalent interactions with the analytes. However, several systems utilize COPs or COFs as support systems or anchor points, so that the actual sensing process happens at another moiety, e.g., transition metal ion, or DNA probe. This is an interesting approach scarcely seen in the detection of other analytes, likely because of the need for extreme levels of specificity in biological systems. These examples demonstrate that covalent polymeric materials can serve various purposes in sensing systems. In addition to being direct responders to analytes, they also have other favorable properties such as porosity, structural robustness, and ability to form noncovalent interactions and can therefore serve as support, anchoring points, or quenchers.

## Sensors for pH

pH sensing is important in biomedical, clinical, environmental, and industrial process control.^77^ The most accurate method of measuring pH of a solution is undoubtedly using a well-calibrated pH electrode, but alternative methods of less accurate but faster and cheaper detection are of interest to the scientific community. In this section, we discuss COP- and COF-based pH sensors. Most of them contain a specific moiety, such as an imidazole, a hydroxyquinoline, or a ketoenamine unit, which can be reversibly protonated in acidic pH. Typically, the protonation prevents the electron transfer and induces a change in the fluorescence properties of the materials. Two strategies of pH sensor design prevail the literature: (i) known small-molecule pH-responsive molecules are integrated into polymeric scaffolds, or (ii) specific protonatable chemical functionalities within the framework are utilized.

**Figure 6:**
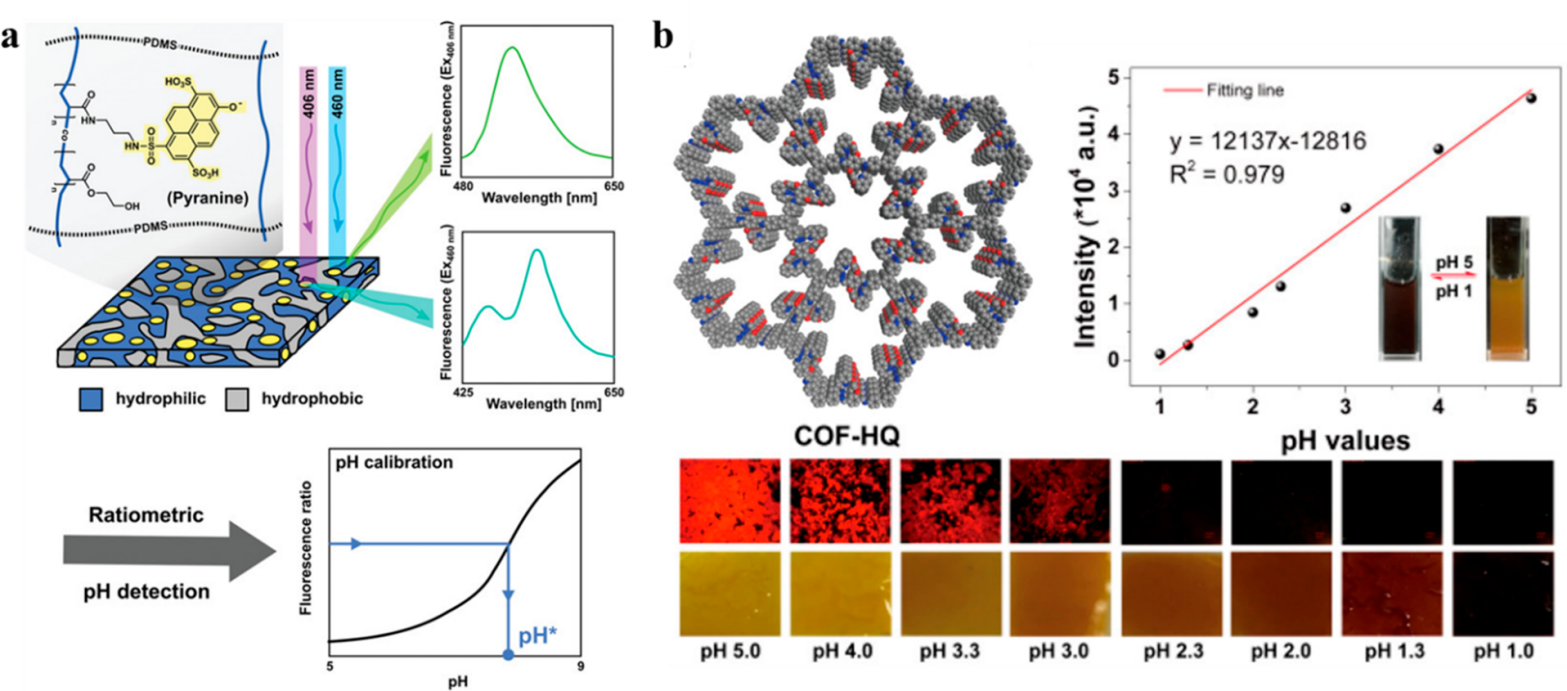
(a) Preparation of ratiometric pH sensor based on PyrAPCN with dual fluorescence. Adapted with permission from ref (79). Copyright 2019 Wiley-VCH Verlag GmbH & Co. KGaA, Weinheim. (b) Structure of COF-HQ, its pH calibration curve, and fluorescence and optical images of the material after exposure to acidic solutions with specific pH levels. Reproduced with permission from ref (82). Copyright 2018 American Chemical Society.

COPs that serve as pH sensors commonly incorporate a fluorescent monomer that can be reversibly protonated, thereby changing its fluorescence signal. Two such moieties were coumarin-based dyes^78^ and pyranine, a known photostable ratiometric pH sensing small molecule.^79^ Coumarin COPs exhibited a decrease in the fluorescence intensity with an increasing pH in the range from 10 to 13.6. This can be rationalized with the structures of the coumarin dyes, which contain an imidazole ring. In neutral and near neutral environments, this ring is protonated and the material exhibits strong fluorescence. As the pH starts to increase in the highly basic level, the proton is gradually lost and the fluorescence signal diminishes. Once deprotonated, the imidazole anion is able to quench the coumarin fluorescence by transferring an electron to the coumarin through PET. In contrast, pyranine in PyrAPCN has two excitation wavelengths (406 and 460 nm) that respond differently to the changes in pH. With increasing pH levels, the emission of the 406 nm excitation increases, whereas that of the 460 nm excitation decreases. Using the ratio of the emission intensities at the two wavelengths enables more precise determination of pH in the range of 5–9 pH units, which is particularly relevant to biological environments (Figure 6a). Increasing pH gradually deprotonates the phenolic hydroxyl and sulfonic acid groups in the pyranine molecule, which are, similar to the imidazole anion above, now able to transfer an electron to the pyrene core through PET.

In the realm of COFs, materials typically do not incorporate moieties that are pH sensors on the monomer level but rather rely on reversible protonation of the linkages that join the building blocks together. For instance, the first example of a pH sensor based on COFs used a Schiff base reaction along with β-ketoenamine formation in COF-JLU4.^80^ This material served as a pH sensor for a broad pH range from 0.9 to 13.0 and exploited its protonatable nitrogen atoms in the β-ketoenamine linkages to induce changes in the fluorescence signal at 428 nm. Above pH 9, the fluorescence intensity gradually decreased due to β-ketoenamine nitrogen deprotonation, whereas below pH 4.5, it blue-shifted and increased with the lowering of the pH as a result of nitrogen protonation. Similarly, COF-TP relied on the protonation of its imine linkages to cause fluorescence quenching with pH decreasing from 6 to 0.^81^

In contrast, 8-hydroxyquinoline-decorated imine COF-HQ relied on a simple protonation of the pyridine unit of 8-hydroxyquinoline. In the protonated state, 8-hydroxyquinoline blocked the π–π* charge transfer interaction from the electron-rich phenol ring to the electron-deficient pyridine and quenched the fluorescence signal (Figure 6b).^82^ This sensor could detect pH in the range from 1 to 5 by the means of fluorescence quenching with decreasing pH, as well as colorimetrically. COF-HQ is as reversible as COF-JLU4 since its fluorescent and colorimetric modes of detection can be restored for at least five cycles.

Integrating known pH sensing molecules into covalent networks contributes to easy recovery and regeneration of the sensor compared to free small molecules. However, these moieties may be difficult to functionalize with groups that can participate in polymerization reactions or may contain functionalities that require protecting groups before polymerization can be carried out. Therefore, strategies that rely on the reversible protonation of COP/COF linkages or protonatable functionalities in the pores may be easier and cheaper to implement. The environment in which these materials are to be used as pH sensors must also be carefully evaluated. It is possible that ions other than hydrogen protons, for instance metal ions, would bind to these sites and potentially hamper the efficiency of the pH sensors.

## Sensors for Solvents and Volatile Organic Compounds

VOCs are atmospheric contaminants released in industrial processes and from transportation vehicles and present in paints, rubber, plastics, solvents, and lubricants. They have been associated with different kinds of irritations and even cancer, so sensors for their detection are highly desired. The examples from the literature in this subsection can be divided into three categories, (i) general VOC sensors, (ii) sensors for specific VOCs, and (iii) water sensors.

A general sensor able to distinguish nitro group-containing VOCs from other types of VOCs was composed of triazine and spirobifluorene subunits and was synthesized by simultaneous Friedel–Crafts and Scholl coupling with different ratios of the reacting monomers.^83^ Sbf-TMP@4:2 was probed for the detection of 20 different volatile organic solvents, and the emission peak at ∼590 nm was quenched in the presence of nitro compounds (e.g., nitromethane) or enhanced upon the addition of other VOCs (e.g., 1,4-dioxane, toluene, acetone, and chloroform). This difference could be explained by the relative positions of the conduction band of Sbf-TMP@4:2 and the LUMO of the analytes. The material’s conduction band is higher than the LUMO of the electron-deficient nitro compounds, so electrons flow from the COP to the analyte, which causes quenching. In contrast, the LUMOs of the other analytes are higher than the conduction band of the COP, so the electrons flow in the opposite direction and the fluorescence signal is enhanced (Table 5). Another COF composed of electron-deficient triazine moieties and electron-rich phenyl and fused phenyl rings was able to distinguish between electron-deficient solvents and the electron-rich ones.^66^ The electron donating VOCs (e.g., toluene, chlorobenzene, and mesitylene) caused red-shifting of the exciplex emission by forming a charge transfer complex, in which electrons were donated by the COF to the VOC.

**Table 5:** COPs and COFs Used as VOC and Solvent Sensors

material	solvent analyte	sensing mechanism	LOD	ref
Sbf-TMP@4:2	nitro compounds	quenching	n/a	(83)
TFPC-NDA COF	electron-donating VOCs	exciplex and CT complex formation	n/a	(66)
TB-TZ-COP	1,4-dioxane	ICT (enhancement)	22.2 ppm	(84)
DhaTab-COF-EuIL	acetone	PET (quenching)	1%	(85)
PTMSDPA	water	deswelling-induced quenching	n/a	(86)
TzDa	water	ICT, ESIPT	0.006–0.085%	(87)

**Figure 7:**
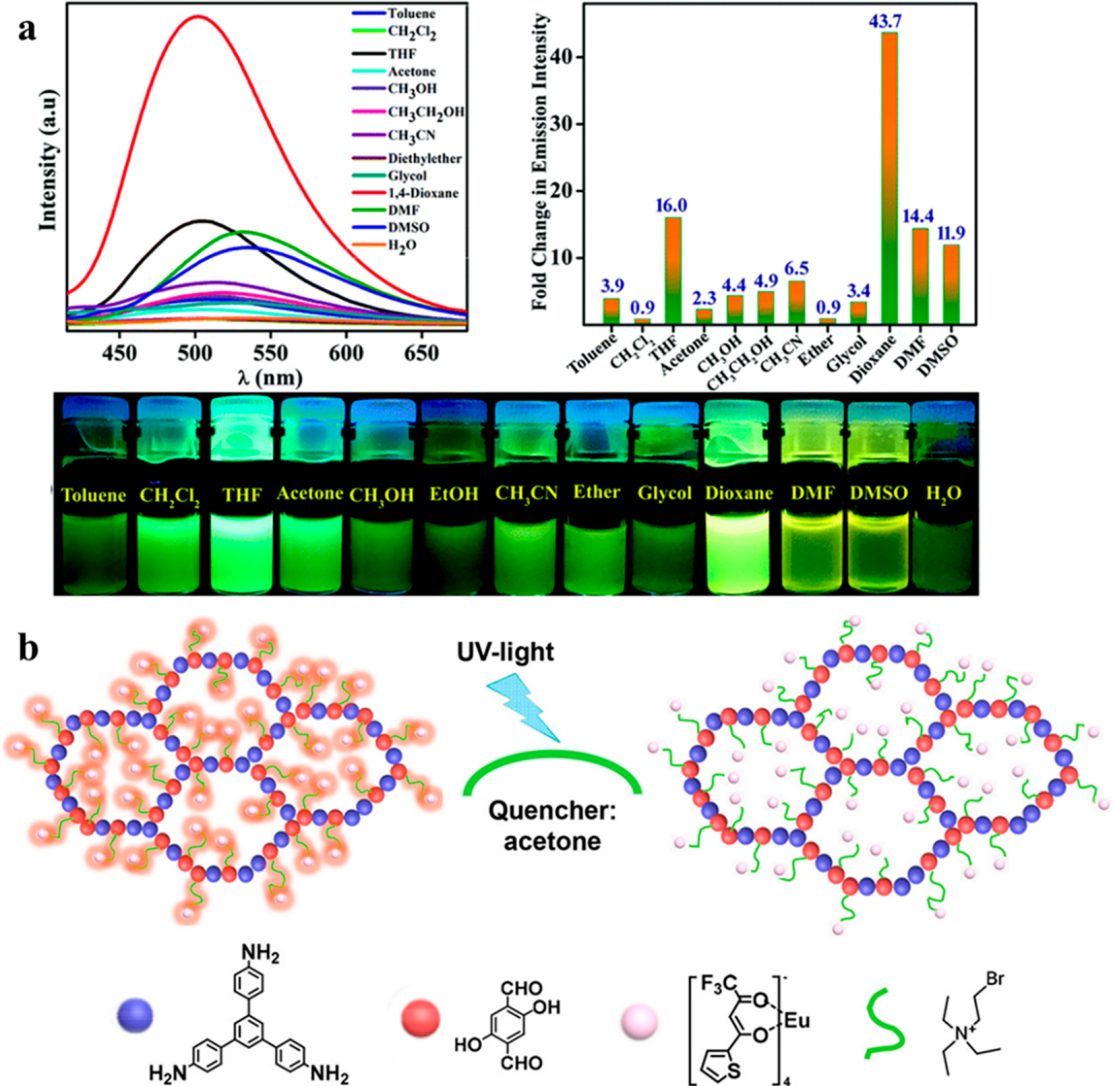
Sensors for various solvents. (a) Emission spectra (λ_ex_ = 360 nm) of TB-TZ-COP in different solvents and the corresponding fold change in emission intensity with respect to its emission in H_2_O along with optical photographs of TB-TZ-COP dispersed in different solvents taken under UV light. Reproduced from ref (84) with permission from The Royal Society of Chemistry. Copyright 2019. (b) Structure and acetone sensing operating principle of DhaTab-COF-EuIL. Reproduced with permission from ref (85). Copyright 2019 American Chemical Society.

COPs and COFs have been investigated as sensors for specific volatile solvents such as 1,4-dioxane^84^ and acetone.^85^ An acetone sensor TB-TZ-COP (Figure 7a) was constructed through imide chemistry based on the amino-1,8-naphthalimide Tröger’s base, a molecule known for its fluorescence due to the ICT mechanism. TB-TZ-COP dispersed in 1,4-dioxane showed a dramatic 44% enhancement of fluorescence upon excitation at 360 nm, while the other solvents showed only modest enhancement (THF, 16%) or caused red-shifting due to polarity of the solvent that stabilized the excited fluorophore. This effect was explained computationally by an electron transfer from the high-energy LUMO of 1,4-dioxane to the lower-energy LUMO of the sensor. The acetone sensor DhaTab-COF-EuIL was likewise selective for the target analyte among 8 tested solvents as its UV spectrum only overlapped with that of acetone. Upon excitation, a competition for adsorption between the two components arises and PET from the material to the solvent and subsequent quenching result (Figure 7b).^85^

Finally, in addition to sensing organic solvents, some applications require an efficient sensor for water, for instance the production of dry solvents needed in organic synthesis. Such sensors have been developed on the basis of a range of principles. For example, poly[1-phenyl-2-(*p*-trimethylsilyl)phenylacetylene] (PTMSDPA) swells in an organic solvent such as ethanol as a result of van der Waals interactions.^86^ When even a small amount of water is added to the system, ethanol forms a hydrogen bond with water and dissociates from PTMSDPA. This causes deswelling of the material, ultimately leading to fluorescence quenching. The stronger the ability of the organic solvent to form hydrogen bonding with water, the more pronounced is the deswelling-induced quenching effect. In contrast, TzDa COF contains the triazine and 2,5-dihydroxyterephthalaldehyde subunits, which both form hydrogen bonding with trace water.^87^ Once this interaction forms, ICT from the phenyl to the triazine core is prevented, resulting in the fluorescence quenching at 500 nm. Furthermore, the hydroxyl group on the terephthalaldehyde no longer forms a hydrogen bond with the imine bond of the COF but rather with a water molecule, thereby preventing the excited-state intramolecular proton transfer (ESIPT) and causing signal enhancement at 590 nm. Similar behavior was obtained for various organic solvents (isopropanol, acetone, THF, ethyl acetate, and ethanol), with LODs between 0.006 and 0.085%.

As previously mentioned for pH sensors, the environment in which the sensor is meant to operate is of immense importance. While the majority of the literature reports delineate the mechanisms of sensor operation and their performance, they rarely look at the specific potential uses of the materials. The use of TB-TZ-COP as a 1,4-dioxane sensor, however, was also studied in the presence of ethylene glycol, which is the starting material for the synthesis of 1,4-dioxane. Therefore, this sensor could be used specifically to study the conversion of ethylene glycol into 1,4-dioxane in an industrial setting. TB-TZ-COP was able to detect 1,4-dioxane in ethylene glycol even at the very low concentration of 22.2 ppm. In future sensor studies, similar considerations of potential interferences should be investigated.

### Iodine

Radioactive iodine is a volatile solid produced in nuclear power plants as a byproduct of the fission of uranium and in detonation of atomic weapons. It is a carcinogenic molecule with radioactive isotopes I^129^ and I^131^ with half-lives of up to 15.7 million years.^88^ Detecting leakages of such toxic species is essential to ensure a safe energy supply.^89^ Several COPs have been developed for remediation of iodine emissions^90−92^ as well as for its detection through fluorescence. Friedel–Crafts^93−95^ and related coupling reactions^96^ have often been used in generating COP sensors for iodine because they can easily generate fully sp^2^-conjugated systems that are fluorescent in nature and possess a high affinity for iodine (dipole–dipole interactions, ionic interactions with polyiodides). Triazine,^97^ tetraphenyl ethylene,^98^ and pyrazine^99^ moieties have all been utilized in the synthesis of COPs through Friedel–Crafts coupling (Table 6). The materials exhibited strong fluorescence in specific solvents, which was quenched upon the addition of I_2_ through the PET mechanism: the excitation light promoted the electron in the COP from the HOMO to the LUMO orbital, from where it was transferred to the LUMO of electron-deficient iodine. The limits of detection were generally the lowest in tetraphenyl ethylene-based systems (2.98 pM and 0.296 pM),^98^ followed by the pyrazine (24.7 pM and 0.136 nM)^99^ and triazine-based systems (80.5 pM and 1.56 nM).^97^ The Stern–Volmer quenching constants were likewise the highest for the tetraphenyl ethylene systems (1.53 × 10^5^ M^–1^ and 9.07 × 10^4^ M^–1^),^98^ and on the order of 10^3^ M^–1^ for the pyrazine- and triazine-containing COPs.^97,99^

**Table 6:** COPs Used as Iodine Sensors

material	mechanism	*K*_SV_ (M^–1^)	LOD	ref
TS-TAD	PET	5.76 × 10^3^	80.5 pM	(97)
TS-TADP	PET	5.59 × 10^3^	1.56 nM	(97)
TTTAT	PET	1.53 × 10^5^	2.98 pM	(98)
TTDAT	PET	9.07 × 10^4^	0.296 pM	(98)
TDPz	PET	3.76 × 10^3^	24.7 pM	(99)
TTDPz	PET	1.10 × 10^3^	0.136 nM	(99)
PTThP-2	static and dynamic quenching	1.99 × 10^3^	75.4 nM	(96)
PTThP-3	static and dynamic quenching	5.09 × 10^3^	29.5 nM	(96)
PCPP	quenching	1.4 × 10^5^	0.314 pM	(100)
TDPA	PET	1.85 × 10^4^	16.2 pM	(94)
TTPBTA	PET	6.56 × 10^4^	6.86 pM	(94)
TDPDB	static and dynamic quenching	5.83 × 10^4^	2.57 pM	(93)
TTPA	PET	2.38 × 10^4^	32.2 pM	(95)
TTDATA	PET	4.33 × 10^2^	n/a	(95)
TTMDATA	PET	7.31 × 10^2^	n/a	(95)

An alternative to Friedel–Crafts reaction, trimerization leading to the formation of the triazine ring from the nitrile-group-containing starting materials yielded the best material reported so far for iodine sensing in terms of the LOD (0.314 pM) and the *K*_SV_ constant (1.4 × 10^5^ M^–1^).^100^ The mechanism for this adsorption stems from the large energy difference of 2.926 eV between the LUMO of I_2_ and the LUMO of PCPP. Despite the plethora of COP-based iodine sensors, to the best of our knowledge, no fluorescent COF-based sensor has been developed for iodine.

### Enantiomers

Enantiomeric sensing is of high interest for monitoring production processes of various biologically relevant molecules as well as for medical diagnostics and biotechnology.^101^ The disaster with thalidomide, a drug often taken by pregnant women with morning sickness, that resulted in thousands of babies born with limb malformations between 1957 and 1961, has drawn attention to chirality and its effects in drug molecules.^102^ We note two strategies for synthesizing covalent network sensors for enantiomers. The first one takes advantage of the different abilities of the d- and l-enantiomers of the analytes to from interactions with the synthesized COPs without using chiral building blocks for the material synthesis.^103^ If an intermolecular interaction is formed, the fluorescence signal is altered. An alternative strategy to enantiomeric sensing in COFs is the incorporation of chiral building blocks into the fluorescent material, which then naturally responds differently to the d- and l-forms of the analytes.

**Figure 8:**
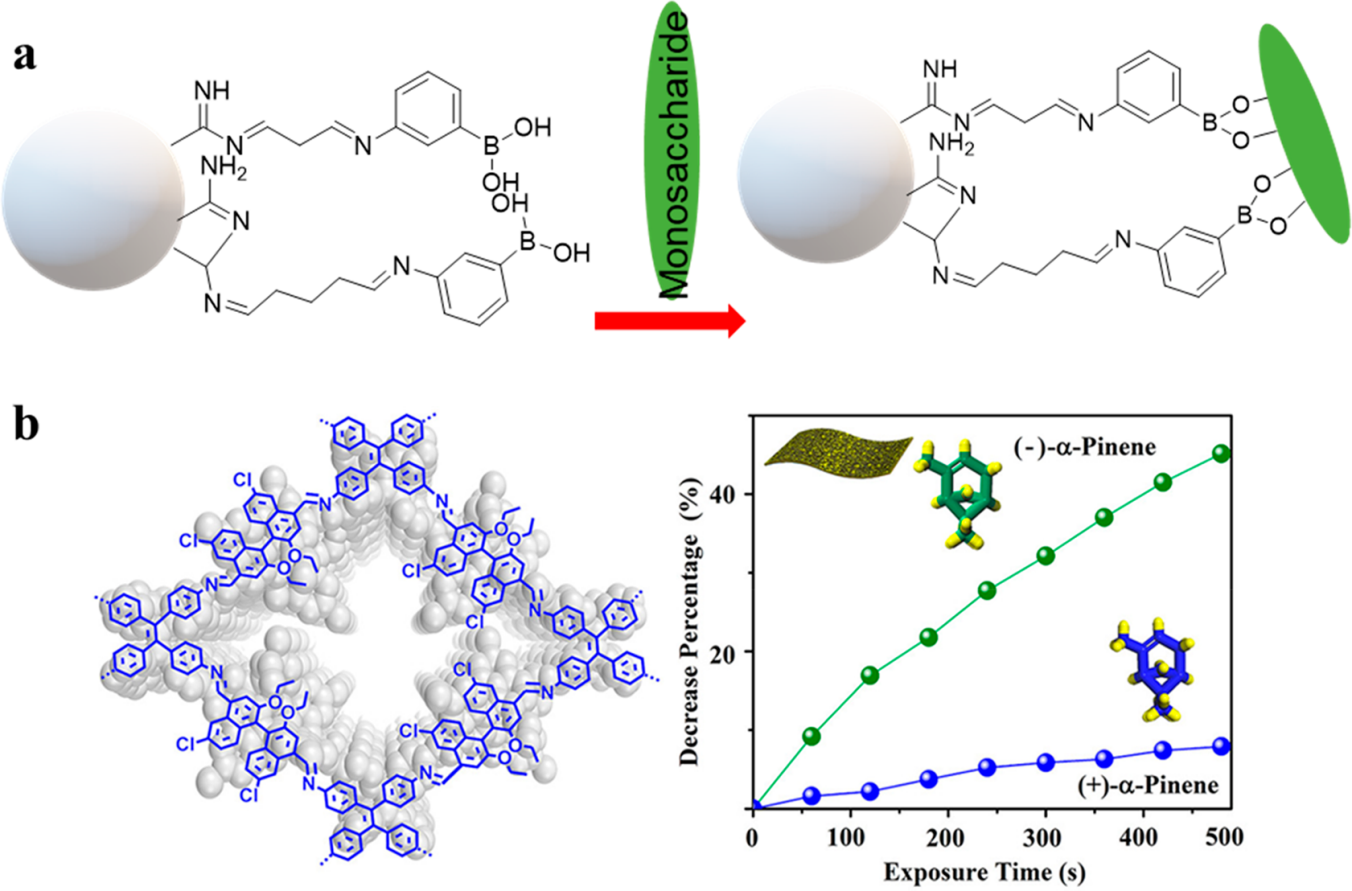
Sensors for chiral molecules. (a) Mechanism of B-PAN for sensing d- and l-monosaccharides.^103^ (b) Structure of CCOF7 and a graph showing its selective (−)-α-pinene detection over (+)-α-pinene. Reproduced with permission from ref (104). Copyright 2019 American Chemical Society.

The first of the two strategies was utilized in monosaccharide enantiomeric sensing in polyacrylonitrile nanoparticles.^103^ To achieve enantioselectivity, these nanoparticles were postsynthetically modified in three steps: first, amidine groups were introduced through the Pinner synthesis; second, Schiff base was synthesized by adding glutaraldehyde to the amidine-terminated nanoparticles; third, the Schiff base was reacted with 4-aminophenylboronic acid to create boronic functionalities at the outer layer (Figure 8a). The modified B-PAN nanoparticles had a dramatically enhanced fluorescence upon excitation at 300 nm, likely as a result of PET between the Schiff base and the phenylboronic acid (Table 7). The material was exposed to different d- and l-monosaccharides and it was observed that the d-enantiomers induced a more pronounced enhancement of the fluorescence signal in the case of glucose and galactose. This enantioselectivity stems from the intramolecular hydrogen bonding, which develops between the imine and amine groups of B-PAN. Further, the protonated tertiary nitrogen and the Lewis acidic ring oxygen form a hydrogen bond, leading to enantiomeric specificity in a material that contains no chiral centers.

**Table 7:** COPs and COFs Employed in Enantiomer Sensing

material	analyte	sensing mechanism	*K*_SV_ (M^–1^)	enantioselectivity	ref
B-PAN	monosaccharides	PET	n/a	n/a	(103)
CCOF7	(−)-(α)-pinene	static quenching	1348	3.49	(104)
CCOF17	d-phenylglycinol	static enhancement	n/a	14.72	(105)
CCOF17	d-phenylalaninol	static enhancement	n/a	12.85	(105)
CCOF17	d-tryptophanol	static quenching	820.5	2.41	(105)

The second strategy was implemented in two recent reports of COFs. One of them contained 1,1′-bi-2-naphthol (BINOL), which is a frequently used source of chirality in organic synthesis and materials science as well as a fluorophore (Figure 8b).^104^ The imine-linked CCOF7 was exfoliated into sheets with a thickness of ∼4 nm. These nanosheets with chiral pores and free ethoxy groups were then investigated for enantiomeric sensing of d- and l-forms of various organic vapors (α-pinene, limonene, fenchone, carvone, and terpinen-4-ol). The addition of any of the chiral vapors induced static quenching, generally with the (−)-enantiomers exhibiting more pronounced and faster quenching. For example, (−)-(α)-pinene quenched the fluorescence faster than the corresponding (+)-enantiomer with *K*_SV_ values of 1348 and 395 M^–1^, respectively. The reported quenching ratios [QR = *K*_sv_(−)/*K*_sv_(+)] were in the range of 1.2–3.49, indicating enantioselectivity for all five tested organic vapors.

In the second report by the same group, two crown ether-based COFs, CCOF17 and CCOF18, synthesized through Knoevenagel condensation were turned chiral following the reduction of the double bonds in the -C=C–CN linkages.^105^ The cavity of the macrocycles in the core could accommodate the amino groups of the three tested chiral amino alcohols. Phenylglycinol and phenylalaninol enhanced the fluorescence emission by forming a crown ether–amino alcohol adduct, which weakened the π–π interactions between the COF layers. In contrast, tryptophanol formed not only the adduct with crown ether groups but also π–π interactions and hydrogen bonds with the COFs through its indole rings and indole NH groups, respectively. This led to the quenching of the fluorescence signal.

While enantiomeric sensing is likely one of the most challenging kinds of chemical sensing, the tunable nature of COFs has proven particularly beneficial. The preparation of these sensors is a daunting task that either requires multiple postsynthetic modifications or a challenging preparation of the chiral monomers. Nonetheless, the alternatives are few and are based on biological macromolecules that pose additional challenges in handling, storing, and utilization.^106^ For these reasons, COFs are possibly some of the most promising types of enantiomeric sensors currently available. However, their direct utilization for compounds of interest to pharmaceutical and other industries is currently lacking.

## Sensors for Ammonia and Amines

Amines are used in numerous industries, including chemical, dye, pharmaceutical, military, and food industry.^107^ While low molecular weight amines, such as ethylamine, are harmful for the aquatic environment but only mildly toxic to humans, higher order amines have been linked to respiratory, neural, and cardiovascular damage in humans or to cancer. Colorimetric COP sensors for ammonia and amines have been studied in the past,^108−110^ with some sensor materials changing color in response to the chemical reduction induced by ammonia,^109^ or as a result of charge transfer interaction between the electron-deficient COP and the electron-rich amines.^110^ However, more recently fluorescence-based sensors have been prioritized on account of their sensitivity. Fluorescent COPs and COFs can detect amines in three different ways: (i) by reacting with free NH_2_ groups of the primary amines,^111^ (ii) by forming hydrogen bonding with hydrogen and/or nitrogen atoms of the amine groups,^112^ or (iii) by accepting an electron pair directly from the electron-rich amine groups (doping).^113,114^

The Suzuki-coupled P7-COP sensor contained aliphatic side chains and aldehyde and trifluoroacetyl side functional groups.^111^ These reticularly chosen aldehyde groups chemically reacted with primary amines through Schiff base formation while the trifluoroacetyl groups formed zwitterions or hemiaminals with organic amines. As a result, P7 responded differently to different types of amines: primary alkyl amines blue-shifted the emission from 525 to 435 nm, secondary alkyl amines blue-shifted the emission to 475 nm, and aromatic amines quenched the fluorescence at 525 nm. The same behavior was also observed in mixtures of amine vapors. The lowest LOD was determined for *o*-toluidine in the ppt range (Table 8).

**Table 8:** COPs and COFs Used as Sensors for Various Amines and Ammonia

material	amine analyte	sensing mechanism	LOD	*K*_SV_ (M^–1^)	ref.
F-CTF-3	phenylamine	static quenching	11.7 nM	8.01 × 10^5^	(112)
F-CTF-3	*p*-phenylenediamine	static quenching	1.47 nM	6.36 × 10^6^	(112)
F-CTF-3	1-naphthylamine	static quenching	26.2 nM	3.57 × 10^5^	(112)
P7-COP	*n*-propyl amine	PET	114 ppm	n/a	(111)
P7-COP	*n*-hexylamine	PET	4.0 ppm	n/a	(111)
P7-COP	diethylamine	PET	190 ppm	n/a	(111)
P7-COP	dipropylamine	PET	0.036 ppb	n/a	(111)
P7-COP	aniline	PET	0.52 ppb	n/a	(111)
P7-COP	*o*-toluidine	PET	0.55 ppt	n/a	(111)
COP-1	NH_3_	ICT	5.9 × 10^–4^	n/a	(116)
P1	*p*-phenylenediamine	PET (quenching)	171 nM	n/a	(115)
P2	aniline	PET (quenching)	81 nM	n/a	(115)
P2	triethylamine	turn-on fluorescence	43 nM	n/a	(115)
TPE-Ph COF	NH_3_	AIE	n/a	4.44 × 10^5^	(117)

**Figure 9:**
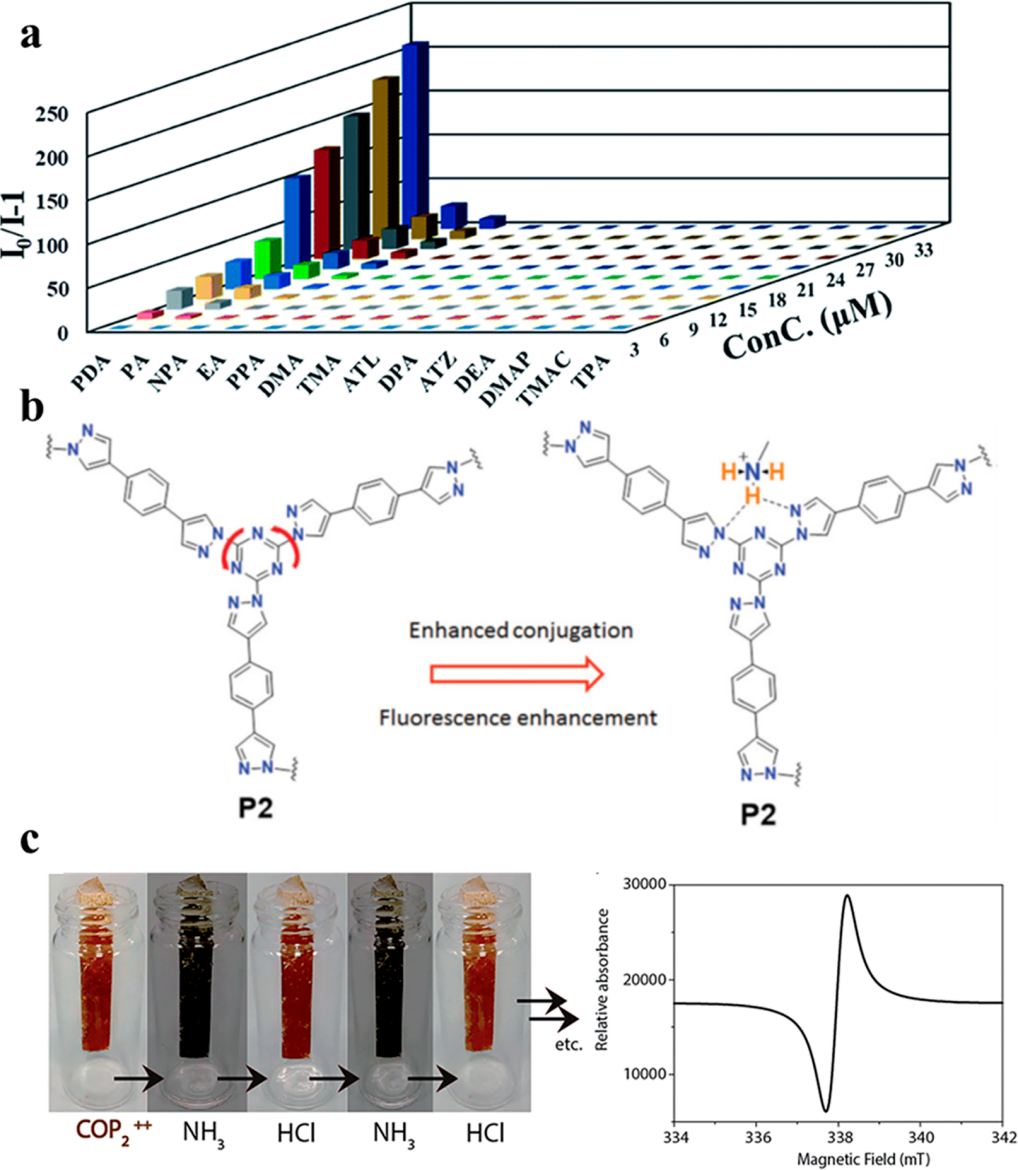
(a) Stern–Volmer plots of 14 different amines tested as analytes for F-CTF-3. PDA = *p*-phenylenediamine; PA = phenylamine; NPA = 1-naphthylamine; EA = ethylamine; PPA = *n*-propylamine; DMA = dimethylamine; TMA = trimethylamine; ATL = amitrole; DPA = diphenylamine; ATZ = 5-amino-1*H*-tetrazol; DEA = diethylamine; DMAP = 4-dimethylaminopyridine; TMAC = tetramethylammonium chloride; TPA = triphenylamine. Reproduced from ref (112) with permission from The Royal Society of Chemistry. Copyright 2020. (b) Sensing mechanism between protonated aliphatic amines and pyrazole rings of P2 leads to conjugation enhancement and turns on the fluorescence signal. Reproduced from ref (115) with permission from The Royal Society of Chemistry. Copyright 2020. (c) Reversibility of NH_3_ sensing with COP2^++^. Sorption of NH_3_ induces the formation of radical cationic viologen species in the COP, as demonstrated by an EPR spectrum. Reproduced from ref (109) with permission from The Royal Society of Chemistry. Copyright 2016.

Amines contain nitrogen and hydrogen atoms that can form hydrogen bonds with a range of functionalities in covalent polymeric materials, either primary linkages, nonreacting functional groups, or postsynthetically introduced moieties. The development of such a network of hydrogen bonds affects the fluorescence properties and is useful in sensing applications. For example, in thiadiazole-based covalent triazine nanosheets F-CTF-3 the main mechanism at play is static quenching that stems from the ground-state nonfluorescent complex formed through hydrogen bonding between the polymer’s thiazoles and the analyte’s NH_2_ groups. This hydrogen bonding interaction is particularly pronounced with *p*-phenylenediamine, and control experiments show that the higher number of amino groups induce more effective fluorescence quenching (Figure 9a).^112^ F-CTF-3 was not only remarkably selective, but it also exhibited record-low LOD for phenylamine and *p*-phenylenediamine in water. A similar mechanism was also observed in the conjugated polymer P1 synthesized from triazine and pyrazole–benzothiadiazole–pyrazole subunits.^115^ Insensitivity to aliphatic amines was explained by the protonation state of the amines. Aliphatic amines are typically protonated at neutral pH, while aromatic amines are not. Therefore, only the latter can transfer the photoinduced electron to the excited state of the polymer and induce fluorescence quenching.

The formation of such a hydrogen bonding network can also have a conjugative effect, leading to turn-on fluorescence. Primary aliphatic amines, which are typically protonated at neutral pH, can extend the conjugative effect on pyrazole rings in P2 (Figure 9b).^115^ Aromatic primary amines are not able to do so at neutral pH because they have lower p*K*_a_ values than aliphatic amines. Instead, they prefer to engage in PET by transferring an electron from the lone pair on the amino group to the polymer.

Finally, NH_3_ and amines contain a free electron pair on the nitrogen atom that can be attracted to an electron-deficient moiety in a COP or COF such as a triazine ring. The electron pair can be directly transferred to the electron-deficient unit through doping. Further, n−π interactions can develop between the lone pair of electrons in NH_3_ and the π-electron cloud in the electron-deficient triazine (Figure 9c).^113,114^ An aldol condensation reaction was used to synthesize an olefin-linked microporous fluorescent COP-1 with a triazine core, which was synthesized in bulk as well as on a quartz surface in the form of thin films.^116^ COP-1 was then used as a sensor for HCl and ammonia gases. Upon exposure to HCl, the fluorescence signal was quenched and red-shifted. The addition of NH_3_ reversed the change and caused an enhancement of the fluorescence signal upon excitation at 453 nm. In the realm of COFs, one of the highly emissive boronate-linked materials served as an ammonia sensor, the first COF to operate by the principle of aggregation-induced emission (AIE).^117^ In such materials, the intralayer covalent and interlayer noncovalent interactions restrict rotation and prevent energy dissipation. The boronate linkages of the COF acted as Lewis acids and NH_3_ served as a Lewis base in such a way that the addition of NH_3_ quenched the fluorescence signal upon excitation in toluene.

The detection of amines is mainly needed for environmental applications (e.g., sensing of pollutants in air) and in the biomedical field (sensing inside the cells).^118^ Depending on which avenue the sensor is being developed for, it must meet a different set of requirements. For any kind of biological sensing, the materials would need to undergo a thorough investigation on biocompatibility, particle size distribution, and membrane permeability, etc. For environmental measurements, fixing the materials into membranes or other kinds of supports would need to be attempted. In the current state-of-the-art, basic research has been mostly focused on investigating the mechanisms of amine sensing and has not yet matured into fully fledged sensing platforms for specific uses. The promising early studies with good selectivity and reversibility in most sensors, and increasingly fast response times, however, indicate a huge potential of these amine sensors.

## Sensors for Gases

Porous covalent networks have been primarily studied in the contexts of gas adsorption, storage, or conversion, but a select number of examples also discussed gas sensing. The property of gas acidity can be exploited to cause protonation of various chemical functionalities in the materials and lead to a change in the fluorescence signal. Hydrogen chloride gas (HCl), for instance, is known to interact favorably with nitrogen centers of organic molecules by reversibly protonating them. This means that sensors reported for this gas operate on a similar principle as the pH sensors discussed above. An imine-linked COF TATF-COM that contained triazine subunits in both of its building blocks has been utilized as a chromogenic and fluorescence-based sensor for HCl.^119^ HCl induced quenching of the fluorescence signal observed at 550 nm with the LOD of 2.38 ppb (Table 9). FTIR measurements indicated that the gas interacted with both triazine and imine nitrogen atoms. Similarly, the imine linkages of a carbazole-containing COF BCTB-BCTA became protonated by HCl.^120^ This enhanced the ICT from one carbazole subunit to the imine and resulted in a red shift in fluorescence from 472 to 495 nm. Further addition of HCl also protonated the carbazole nitrogen atoms, which impeded the ICT and produced the quenching of the fluorescence signal at 495 nm, reaching the LOD of 10 nM.

**Table 9:** COPs and COF Used as Sensors for Various Gases

material	gas analyte	sensing mechanism	LOD	*K*_SV_ (M^–1^)	ref
TATF-COM	HCl	quenching	2.38 ppb	n/a	(119)
BCTB-BCTA	HCl	ICT (quenching)	10 nM	n/a	(120)
Polym-H7	CO_2_	quenching	1.3%	n/a	(121)

Carbon dioxide (CO_2_) is also mildly acidic, so its sensing can operate on a similar principle. A fluorescence-based Polym-H7 sensor has been developed to detect CO_2_ down to 1.3% (v/v) levels.^121^ This commercially available polymer is composed of fluorescein *o*-acrylate (a pH indicator), methyl methacrylate (MMA) and hydroxymethyl methacrylate (HEMA) and can be incorporated into films by spin-coating. If present, CO_2_ reacts with tetraoctylammonium hydroxide (TONOH) to form anionic bicarbonate and cationic TON^+^. The fluorescein *o*-acrylate pH indicator in the polymer exhibits two fluorescence spectra, one for its protonated native state and the other for its negatively charged state complexed with TON^+^. On the basis of the ratio of the two, the concentration of CO_2_ can be determined.

From an industrial point of view, the global gas sensors market is estimated at 2.19 billion USD in 2019 with most sensors used for the detection of oxygen, carbon dioxide, and nitric oxide.^122^ However, most of CO_2_ sensors, for instance, rely on parameters other than fluorescence, including capacitance and resistivity, and are based on inorganic materials.^118^ Given that these materials reach good levels of detection and can be synthesized at lower costs than COPs and COFs, it becomes fathomable why few of the latter have been investigated for the sensing of common gases. They may be better suited for the sensing of more intricate chemicals in the gaseous phase, such as chiral α-pinene vapors.

## Sensors for Anions

Among various anions, oxoanions such as CrO_4_^2–^, Cr_2_O_7_^2–^, and MnO_4_^–^ are commonly used oxidants in laboratories and in industry. However, these anions contain heavy metals which can be toxic, so their presence needs to be closely monitored.^109^ For this purpose, the development of sensors for oxoanions is of great importance, although the current survey of the literature suggests that oxoanion removal is more commonly studied than oxoanion sensing.

One strategy of designing heavy metal oxoanion sensors is to synthesize COFs with UV absorption spectra that overlap with those of oxoanions. Such materials are also likely to be selective because nonheavy metal containing oxoanions will absorb light at higher energies. A recently reported 3D TT-COF with a bis(tetraoxacalix[2]arene[2]triazine) core was found to be an efficient sensor for CrO_4_^2–^, Cr_2_O_7_^2–^, and MnO_4_^–^ while not being sensitive to 16 other anions upon excitation at 490 nm.^123^ The LODs for all three anions were ∼0.3 mM, and the Stern–Volmer quenching constants were ∼1.4 × 10^4^ M^–1^ (Table 10). The UV–vis absorbance spectra of TT-COF overlapped in several regions with the spectra of the three anions, so the mechanism of fluorescence quenching was found to be based on absorption competition. When the analytes absorb the excitation energy, they suppress excitation energy transfer to an organic ligand of COF-TT, thereby decreasing the emission intensity or quenching it.

**Table 10:** COPs and COF Used as Anion Sensors

material	anion analyte	sensing mechanism	LOD	*K*_SV_ (M^–1^)	ref
TT-COF	CrO_4_^2–^	ACQ	0.343 mM	1.4 × 10^4^	(123)
TT-COF	Cr_2_O_7_^2–^	ACQ	0.343 mM	1.4 × 10^4^	(123)
TT-COF	MnO_4_^–^	ACQ	0.320 mM	1.5 × 10^4^	(123)
DATG_Cl_	F^–^	PET (quenching)	5 ppb	2.25 × 10^3^	(124)

Another strategy of designing anion sensors is to incorporate specific linkers, such as guanidine that can donate a proton to an anion serving as a Bronsted–Lowry base, into the backbone of the COFs. Pal et al. developed a cationic guanidine-based fluorescent sensor, DATG_Cl_, which responded to strongly basic anions, primarily F^–^, but also OH^–^, SO_4_^2–^, and CO_3_^2–^.^124^ The guanidine linker and, specifically, its protonation state play a major role in the process of detection. Fluoride anion is small, polarizable, and basic enough to abstract a proton from guanidine, which becomes neutral and is now able to transfer an electron to the fluorophore aldehyde in the backbone of DATG_Cl_. DFT calculations on the system showed that the abstraction of a guanidine proton increases the HOMO–LUMO energy so that an electron can be easily transferred to the fluorophore aldehyde via the PET mechanism. Impressively, the LOD for fluoride anions of this system is 5 ppb, which is the lowest ever reported for F^–^ in any class of materials.

## Conclusion and Future Perspectives

In summary, this review discussed the physical basis of fluorescence in covalent polymeric materials and explained how fluorescence spectroscopy can be used to design covalent polymeric sensor materials. It detailed the sensing properties of COPs and COFs for various analytes, including heavy metal ions, explosives, biological molecules, pH, solvents and VOCs, iodine, enantiomers, ammonia and amines, gases, and anions. After a thorough analysis, we conclude that the most common mechanism of fluorescence sensing involves PET, in which a photoexcited electron is transferred from the HOMO or LUMO of the donor to the LUMO of the acceptor, either of which can be the fluorophore. In most reports, the fluorescence intensity is quenched by the addition of the analyte, although several cases of turn-on fluorescence have also been reported and discussed herein. Low LODs for specific analytes in some discussed materials demonstrate the potential of these types of materials for sensing applications; for instance, the most sensitive sensor for iodine among all classes of materials is the PCPP COP.^100^ Moreover, certain COPs and COFs have extraordinarily short response times of as little as 2 s,^31,32^ in addition to being insoluble in water and common organic solvents, which makes separation, purification, and recycling far more efficient than for soluble materials.^10^

In spite of the major advantages of the two classes of materials as sensors, there are also several areas where improvements are needed.

- The majority of reports utilize sensors in organic solvents rather than in water. This might be due to better analyte solubility in organic solvents, better dispersibility in nonaqueous media, or better efficiency of the electron transfer. It is, however, beyond doubt that water is the most common liquid medium in which detection of pollutants, toxic compounds, and other analytes is of interest in practical applications. Therefore, more emphasis should be placed on designing sensors with efficient operation in aqueous media.
- A sensor is only efficient if it can be used multiple times. This ultimately requires developing a procedure for detaching the target analyte, which does not harm the structure and function of the materials, so that their selectivity and sensitivity to the target are preserved and the sensor can be reused. Currently, many reported materials have not been tested for regenerability, or partly or fully lost their detection potency after regeneration. Yet it is essential to explore this aspect of any material considered to be a sensor in real-life applications.
- It is far easier to detect the appearance of a bright signal against a dark background than to detect the disappearance of a bright signal. Therefore, emphasis should be placed on developing the turn-on fluorescence sensors rather than relying primarily on the turn-off materials. This can be achieved in 2D materials whose individual layers are stacked, so no fluorescence is observed, but the addition of the analyte relieves the aggregation-caused quenching.^45^ Alternatively, rapid isomerization may prevent fluorescence of the materials, but the addition of the analyte prevents such isomerization, ultimately turning-on the fluorescence response.^28^
- The majority of the referenced examples detect analytes in suspensions. Few efforts have been made to investigate how they can be used as sensors in the solid state although this approach can afford very promising results. For instance, when an imide-linked COF was tested for nitroaromatics detection in suspension, its fluorescence was quenched by the analytes. However, when the same material was used in a solid-state sensor, the addition of TNP enhanced the fluorescence signal.^63^
- Finally, COPs and COFs are typically composed of anisotropic particles of various dimensions, which can interfere with reproducibility of the observations. Therefore, protocols should be developed to minimize such variations among particles by mechanically grinding them, incorporating them into membranes, or coating them on solid supports.

This analysis of the current state-of-the-art in sensing applications demonstrates that the recent developments and the impressive performance of COPs and COFs sensors justify their further investigation and optimization. It also shows that, in the majority of cases, research has focused on understanding the interplay between the materials’ and the analytes’ structures and their performance in sensorics. Many mechanistic studies and theoretical modeling have also aimed at gaining a thorough understanding of the mechanisms of these fluorescence sensors. What has commonly been overlooked, and where the greatest opportunities await in the future, is tailoring these sensors to very specific applications and investigating all of the external factors that may influence the sensor effectiveness. This involves performing various competition and interference studies, optimizing the physical parameters such as temperature, pressure, and humidity, etc., and building full sensor setups.

## Vocabulary

Covalent organic polymer: a mostly insoluble, often porous polymer with building blocks linked to each other through covalent bonds
Covalent organic framework: a crystalline porous extended structure characterized by light elements and dynamic covalent bonding between the building blocks
Fluorescence spectroscopy: an analytical technique that detects the light emission from a sample promoted from the ground electronic to the excited electronic state by the incident excitation light
Analyte: a substance being identified (in the context of sensorics, often the moiety that induces changes in the fluorescence spectrum of the sensor)
Fluorescence quenching: a common type of fluorescence mechanism where the fluorescence intensity of a substance or material decreases as a result of transferring energy to another molecule

## Abbreviations

ACQ: aggregation-caused quenching
AIE: aggregation-induced emission
ATL: amitrole
ATZ: 5-amino-1*H*-tetrazol
BINOL: 1,1′-bi-2-naphthol
BODIPY: 5,5-difluoro-1,3,7,9-tetramethyl-10-phenyl-5*H*-dipyrrolodiazaborinine
CHEQ: chelation enhanced quenching
COF: covalent organic framework
COP: covalent organic polymer
DEA: diethylamine
DMA: dimethylamine
DMAP: 4-(dimethylamino)pyridine
DMF: *N*,*N*-dimethylformamide
DNA: deoxyribonucleic acid
DNP: 2,6-dinitrophenol
DNT: 2,6-dinitrotoluene
DPA: diphenylamine
EA: ethylamine
EDTA: ethylenediaminetetraacetic acid
EE: electron exchange
EPR: electron paramagnetic resonance
ESA: excited-state absorption
ESIPT: excited-state intramolecular proton transfer
FRET: Förster resonance energy transfer
FTIR: Fourier transform infrared
HEMA: hydroxymethyl methacrylate
HOMO: highest occupied molecular orbital
IC: internal conversion
ICT: internal charge transfer
IFE: inner filter effect
ISC: intersystem crossing
K_SV_: Stern–Volmer constant
LOD: limit of detection
LUMO: lowest unoccupied molecular orbital
MMA: methyl methacrylate
MOF: metal–organic framework
NMR: nuclear magnetic resonance
NP: 2-nitrophenol
NPA: 1-naphthylamine
NT: 2-nitrotoluene
PA: phenylamine
PDA: *p*-phenylenediamine
PET: photoelectron transfer
PPA: *n*-propylamine
RET: resonance energy transfer
RNA: ribonucleic acid
THF: tetrahydrofuran
TMA: trimethylamine
TMAC: tetramethylammonium chloride
TNP: 2,4,6-trinitrophenol
TNT: 2,4,6-trinitrotoluene
TPA: triphenylamine
UV: ultraviolet
VOCs: volatile organic compounds
WHO: World Health Organization
XPS: X-ray photoelectron spectroscopy
